# Vaccine preferences driving vaccine-decision making of different target groups: a systematic review of choice-based experiments

**DOI:** 10.1186/s12879-021-06398-9

**Published:** 2021-08-28

**Authors:** Marilyn Emma Diks, Mickael Hiligsmann, Ingeborg Maria van der Putten

**Affiliations:** 1 Faculty of Health Medicine and Life Sciences, Maastricht University, Universiteitssingel 40, 6229 Maastricht, Netherlands; 2 Department of Health Services Research, Care and Public Health Research Institute (CAPHRI), Maastricht University, Duboisdomein 30, 6229 Maastricht, Netherlands

**Keywords:** Discrete choice experiment, Conjoint analysis, Stated preferences, Vaccine behaviour, Vaccine decision-making, Target groups

## Abstract

**Background:**

Choice-based experiments have been increasingly used to elicit preferences for vaccines and vaccination programs. This study aims to systematically identify and examine choice-based experiments assessing (differences in) vaccine preferences of vaccinees, representatives and health advisors.

**Methods:**

Five electronic databases were searched on choice-based conjoint analysis studies or discrete choice experiments capturing vaccine preferences of children, adolescents, parents, adults and healthcare professionals for attributes of vaccines or vaccine settings up to September 2020. Data was extracted using a standardized form covering all important aspects of choice experiments. A quality assessment was used to assess the validity of studies. Attributes were categorized into outcome, process, cost and other. The importance of attributes was assessed by the frequency of reporting and statistical significance. Results were compared between high-quality studies and lower-quality studies.

**Results:**

A total of 42 studies were included, with the majority conducted in high-income countries after 2010 (resp. *n* = 34 and *n* = 37). Preferences of representatives were studied in nearly half of the studies (47.6%), followed by vaccinees (35.7%) and health advisors (9.5%). Sixteen high-quality studies passed the quality assessment. Outcome- and cost- related attributes such as vaccine effectiveness, vaccine risk, cost and protection duration were most often statistically significant across both target groups, with vaccine effectiveness being the most important. Risks associated with vaccination, such as side effects, were more often statistically significant in studies targeting vaccinees, while cost-related attributes were more often statistically significant in studies of representatives. Process-related attributes such as vaccine accessibility and time were least important across both target groups.

**Conclusion:**

To our knowledge, this is the first systematic review in which vaccine preferences of different target groups were assessed and compared. The same attributes were most important for vaccine decisions of vaccinees and representatives, with only minor differences in level of evidence for vaccine risk and cost. Future research on vaccine preferences of health advisors and/or among target groups in low-resource settings would give insight into the generalizability of current findings.

**Supplementary Information:**

The online version contains supplementary material available at 10.1186/s12879-021-06398-9.

## Background

Within the last decades, the understanding of vaccine decision-making has been expanded. Vaccine decisions are no longer considered as simple binary decisions, but rather as complex multifaceted decisions taken along a continuum [1, 2]. To arrive at a vaccine decision, individuals consider a set of alternatives that are evaluated based on individual needs and interests [3]. Vaccine decisions are, hence, subject to multiple internal and external stimuli, such as personal values [4, 5], information sources [4, 6, 7], social support [8], risk perception, vaccine effectiveness [5, 8] and provider trust [4–6]. As a consequence, various (possibly opposing) values may be assigned to characteristics of vaccine alternatives resulting in a wide range of vaccine preferences and decisions. Moreover, health-related preferences depend on whom it is taken for (i.e. the decision-making role) [9]. For vaccination, the decision could either be taken by the vaccinee or someone else. A vaccinee is defined as an individual to whom a vaccine is administered and who is often involved in vaccine-decision making. However, vaccinees do not necessarily need to draw the actual vaccine decision, and be the decision-maker [10].

Representatives or health advisors may also be entitled to make the decision for the vaccinee. Representatives refer to parents, guardians, relatives and others with formal authority, who decide for instance to vaccinate a child. Health advisors refer to healthcare provides or caregivers (such as family doctors) to whom decision authority is ceded by the vaccinee [9, 10].

A study of Goldstein & Weber [11] indicates that individuals apply different strategies when deciding for themselves or someone else. In line with this, Zikmund-Fisher, Sarr, Fagerlin & Ubel [9] demonstrate substantial variation in treatment preferences between decision-making roles. Medical professionals and parents are for instance more proactive in flu vaccination (i.e. choose to vaccinate) than vaccinees. Additionally, health-related preferences and decisions vary according to the importance of decisions [12]. Particularly in the context of rising vaccine opportunities, global vaccine implementation and the associated rise in vaccine decisions [13, 14], the complexity of and variation in vaccine preferences may increasingly affect vaccine uptake. Therefore, it is important to gain insight into vaccine-related behaviour including preference differences between decision-making groups.

With respect to preferences, a distinction is made between revealed preferences (RP) and stated preferences (SP). While RP focus on current vaccine behaviour and analyse observed choices, SP describe hypothetical vaccine decision contexts and are based on the analysis of individual choices (stated behaviour) between hypothetical alternatives**.** These stated choices are assumed to reflect and comply with decisions in real-life settings and are increasingly applied in health economics to understand the valuation of existing or future vaccines, to forecast (changes in) vaccine behaviour and/or to determine the willingness-to-pay (WTP) for particular alternatives [15–18]. To capture preferences in vaccination, choice-based experiments, such as Discrete Choice Experiments (DCEs) and Conjoint Analyses (CAs), are most often used [19]. Within these experiments individuals are given series of hypothetical vaccine scenarios and asked to choose their preferred scenario from a given choice set (e.g. vaccine A or B) [18]. Each scenario in a choice set is constructed by the same attributes (e.g. vaccine effectiveness, protection duration, side effects), but with varying attribute levels (e.g. effectiveness of 50% vs. 99%). By analysing individuals’ responses to changing level of attributes, attribute trade-off information is obtained and the relative importance of attributes as well as the expected vaccine uptake of current or hypothetical vaccines could be estimated [19].

Despite the growing interest in the use of choice-based experiments in vaccination, limited reviews have been conducted on this topic. Moreover, preceding reviews of SP research mainly focused on preferences for specific vaccines (e.g. HPV vaccine) and was usually restricted to High-Income Countries (HICs) [19–21]. Michaels-Igbokwe MacDonald & Currie [19] published in 2017, for instance, a review on preferences for childhood and adolescent vaccines. However, due to their methodological focus, no conclusions were drawn on vaccine attributes influencing vaccine decisions. Furthermore, no studies nor reviews examined the differences in vaccine preferences between decision-making groups such as vaccinees and representatives. Given the global challenge of vaccine hesitancy [14] and limited effectiveness of policy measures fostering vaccine uptake [22, 23], it is important to gain deeper insight into general preferences for vaccine characteristics as well as differences in vaccine preferences. This will provide an overview of global vaccine preferences and offers the prospect of improving vaccine uptake by creating new and adapting existing policy measures and strategies to the needs of specific target groups. This approach does, hence, not only fit the life-course approach of the European Commission [24, 25], but also the recommendations of the Strategic Group of Experts on Immunization [14] which stressed the need to understand drivers of vaccine decision-making and implement tailored strategies improving vaccine uptake.

Therefore, this study aims to review, summarize and critically assess studies that used choice-based experiments to measure SP in the field of vaccination. In addition, we aimed to identify vaccine attributes influencing vaccine decision-making of specific target groups (i.e. vaccinees, representatives and health advisors) and to examine differences between vaccine preferences of target groups.

## Methods

### Search terms and strategy

To obtain a comprehensive overview of the current SP literature on vaccine decision-making, a systematic review was conducted. Five electronic databases were searched to identify published choice-based experiments capturing vaccine preferences. PubMed, EMBASE, Web of Science, EconLit and CINAHL were searched on the search terms: “vaccin* OR immunis* OR immuniz*” AND “discrete choice OR choice experiment OR DCE OR conjoint analysis OR stated preference” AND “preference”. The strategy was adapted from the review of Michaels-Igbokwe et al. [19]. Subject headings were used if applicable (MeSH terms in PubMed, Emtree terms in EMBASE and CINAHL subject headings in CINAHL). An overview of the search strategy is included in Additional file 1. The search was limited to articles concerning human vaccines and vaccination programs. Studies who met the following inclusion criteria were included in this review: 1) describing a choice-based conjoint analysis study or a DCE; 2) targeting preferences of children, adolescents, parents, adults and/or healthcare professionals or societal preferences for attributes of vaccines or the setting; 3) original scientific research written in English. Studies without a component of choice, such as studies covering methods on time trade-off, ranking or best-worst scaling, were hence not eligible for this study. Moreover, re-analyses were excluded and duplicates were removed manually. Titles and abstracts of identified studies were then screened for relevance. Full texts of relevant studies were assessed for eligibility. Backward and forward snowballing were applied to check for additional studies [26]. Previous reviews on vaccine preferences were also checked for additional studies [17–19, 21, 27, 28]. The search was conducted between April and May 2020. An update to the review was conducted in September 2020. The PRISMA flow diagram and the PRISMA checklist were used to draw this report [29].

### Assessment of included studies

#### Review of study characteristics

A standardized form was used to extract and review data from each included study. This form was in correspondence with templates used by previous reviews [19, 30] and covered topics related to the: a) study characteristics, b) choice task and experimental design, c) conduct, d) analysis, and e) journal and funding. As previous research [19–21] indicated that choice-based experiments use various definitions/terminology for similar attributes, attributes were first divided into four overarching categories: outcomes, process, cost and other. ‘Outcomes’ referred to the results or consequences of administering vaccines. ‘Process’ incorporated the activities related to the delivery and administration of vaccines and ‘cost’ covered the (financial) costs of vaccines. Attributes that could not be grouped under the former three were classified into the category ‘other’. Within all four categories, attributes with shared features were then grouped according to their underlying concept. These groups of attributes were called ‘domains’ and can be regarded as subgroups which allowed a more comprehensive synthesis of results. Data was extracted from full text articles and corresponding supplementary material that was available online. The search, data extraction, review of study characteristics, quality assessment and data comparison were conducted by one reviewer (MD). Eligibility of ambiguous studies and study characteristics were discussed with a second researcher (IvdP). Atlas.ti (version 8.4.4) and spreadsheets of MS Excel were used for the data extraction and quality assessment.

#### Assessment of quality

Prior to the data comparison, the methodological quality of included studies was critically appraised by using the 13-criteria-checklist of Mandeville Lagarde & Hanson [30], which incorporates all key stages DCEs: choice task design, experimental design, conduct and analysis. Full texts of included studies were appraised by allocating scores to each criterion of the checklist. Three answer options (scores) were possible and depended on the presence of items. A score of 0 was assigned to items that were not satisfied, absent or not reported, 0.5 to items that were partly present or satisfied and 1 to items that were present or satisfied [19, 30]. The maximum score was 13 and was associated with a high methodological quality. Accordingly, the minimum score of 0 indicated a low methodological quality. As recommended by the developers of the checklist [30], the quality threshold of 75% was used. A quality score of at least 10 (out of 13) was considered sufficient to be included in the data comparison.

#### Data comparison

A descriptive synthesis was used to indicate the relative importance of individual vaccine attributes for specific target groups. The amount of times that particular vaccine attributes (domains) were reported in studies were counted as well as the amount of times domains were reported statistically significant by the authors (incl. *p*-value threshold or alpha). Main models and overall results were used if available. Subgroup results were used when outcomes were reported for subgroups/classes only (i.e. no overall data). If a study included multiple attributes related to the same domain (e.g. ‘vaccine side effects’ and ‘risk of dosing’ both targeting the domain ‘vaccine risk’), statistical significance was reported for each of the attributes separately. This implies that a study could report statistical significance for a single domain more than once. To ensure a more accurate reflection of domains driving decision-making, the amount of studies reporting statistical significance, for a particular domain, were also stated. If no *p*-value threshold was reported but *p*-values were given, the commonly used threshold of *p* < 0.05 was used [31]. The overall frequency as well as the classification of statistically significant attributes/domains were presented in a tabular summary and were reported for each target group [32].

#### Comparison of high- and lower-quality studies

To determine whether exclusion of lower-quality studies changed findings for any of the target groups (i.e. inferred selection bias), results of only including high-quality studies (quality score ≥ 10) were compared to results of including all eligible studies (quality score 0–13). Face validity was used to determine if and to what extend results were in accordance with each other.

#### Ethics

Before the start of the study, a review protocol was submitted on PROSPERO (ID: 178245). The review was executed as planned/described. No ethical approval of a Medical Review Ethics Committee was needed [33].

## Results

### Search results

**Fig. 1:**
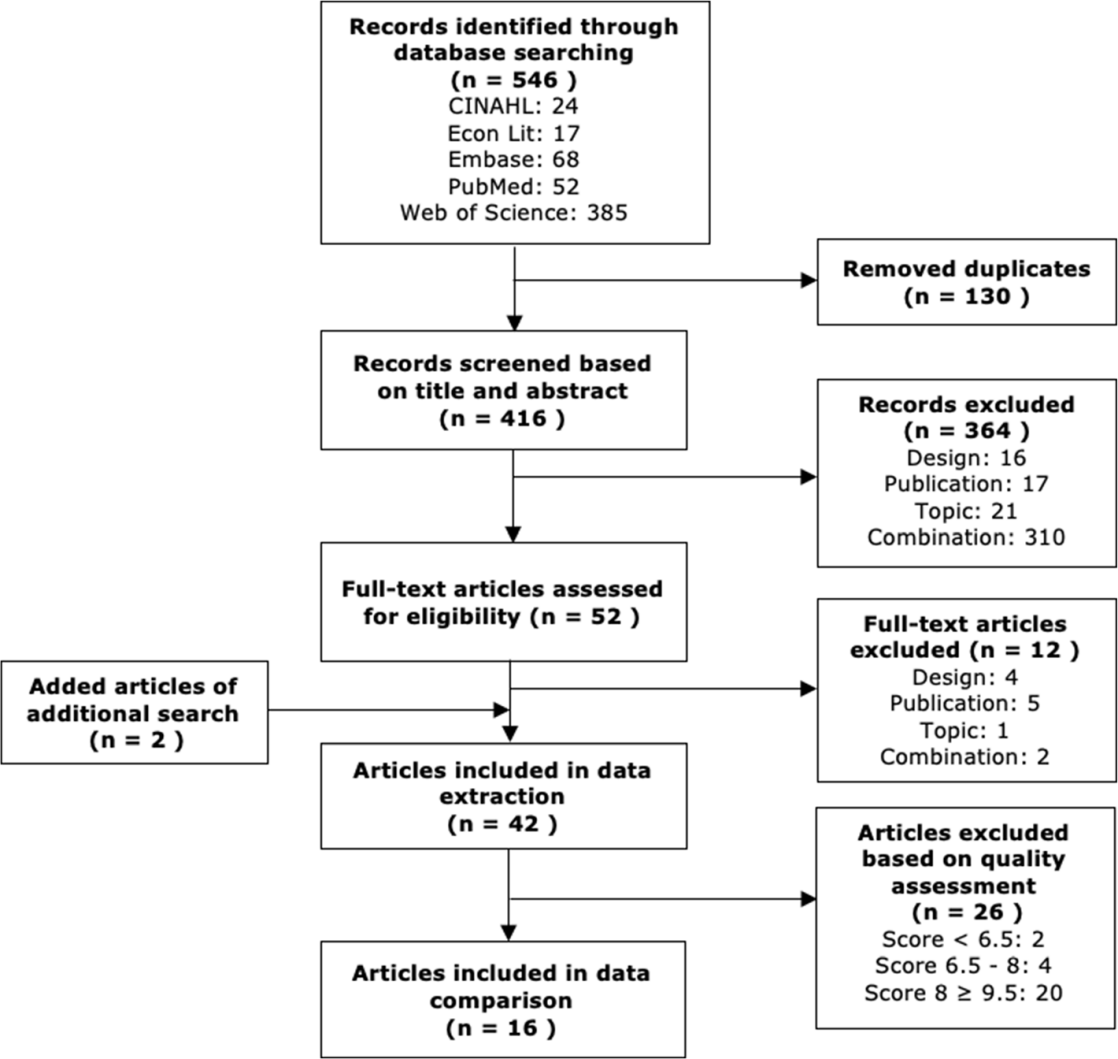
PRISMA flow diagram of choice-based experiments capturing vaccine preferences

In total, 546 records were identified during the primary search. After removal of duplicates, 416 unique records were screened on title and abstract. This resulted in a further removal of 364 records, after which 52 remained left for full-text screening. Reason for removal related to the inappropriateness of the study design, study topic, type of publication (e.g. re-analysis, meeting/conference abstract, erratum) or a combination. During the subsequent searches, two additional articles were obtained (one through snowballing, one through search update). A total of 42 articles were eligible and were included in the review of study characteristics. In addition, 26 articles did not pass the quality assessment as their score was below 10 (see validity assessment). Eventually, sixteen articles were included in the data comparison (Fig. 1).

### Review of study characteristics

Characteristics of all 42 studies were described in this section in order to provide a full overview of the current SP literature on vaccine preferences. A more detailed summary of study characteristics is presented in Additional file 2.

#### General study characteristics

General characteristics of the 42 included studies can be found in Tables 1 and 2. Most choice-based experiments applied a DCE or CA format (resp. 73.8 and 23.8%). The majority of the studies (*n* = 37) were published after 2010: nineteen (45.2%) between 2011 and 2015 and eighteen (42.9%) between 2016 and September 2020 (Table 1). Included studies were conducted in twenty countries mainly spread across Europe (*n* = 25), Asia (*n* = 11) and North America (*n* = 8) (Table 2). More than 80% was conducted in HICs (*n* = 34). Choice experiments were least performed upper and Low-Middle-Income Countries (LMICs). Fourteen existing vaccines or vaccine programs were studied, most commonly HPV and influenza vaccines (Table 2). Vaccine programs referred either to the administration of a course of vaccines (e.g. all childhood vaccines) or to combination vaccines (e.g. Tdap). Preferences of representatives were most often studied (47.6%, Table 1). This target group usually referred to (expectant) parents, guardians or caregivers (*n* = 19), in particular mothers of children aged below 5 (*n* = 9). Vaccinees were targeted in a third of the studies (35.7%). They either focused on the (general) adult population (*n* = 7) or children/adolescents (*n* = 6), especially teenage girls. Preferences of health advisors such as paediatricians were least captured among included studies (9.5%, Table 1). The variety of objectives reported in studies is presented in Table 1.

**Table 1:** General study characteristics of included studies

Aspect	Specification	Number of studies (%)
**Choice based experiment**	ADCE	1 (2.4)
	CA	10 (23.8)
	DCE	31 (73.8)
**Year of publication**	2000–2005	2 (4.8)
	2006–2010	3 (7.1)
	2011–2015	19 (45.2)
	2016–2020 (September)	18 (42.9)
**Target group**^a^	Health advisors	4 (9.5)
	Representatives	20 (47.9)
	Vaccinees	15 (35.7)
	Vaccinees & representatives	3 (7.1)
**Objective**^b^	Assess preferences vaccines, vaccine attributes, vaccine programs	36 (85.7)
	Compare individual DCE	1 (2.4)
	Compare RP with SP	1 (2.4)
	Estimate WTP	15 (35.7)
	Explore variation in preferences across groups	8 (19.0)
	External factors influencing preferences	6 (14.3)
	Identify reason(s) not to vaccinate	1 (2.4)
	Predict vaccine uptake/coverage	11 (26.2)
	Policy recommendations design and/or communication of vaccine programs/strategies	10 (23.8)
	Trade-off vaccine attributes	6 (14.3)

^a^Due to rounding of percentages, the total may not count up to 100%; ^b^As 32 studies included more than objective, the total number of studies exceeds the total amount of included studies (and 100%)

**Table 2:** Number of studies per country and vaccine type

Country	Number of studies (%)^a^	Type of vaccine	Number of studies (%)^a^
Australia	3 (7.1)	Childhood (combination) vaccines	6 (14.3)
Belgium	1 (2.4)	General vaccines	3 (7.1)
Canada	1 (2.4)	Hepatitis B (HepB) vaccine	2 (4.8)
China	3 (7.1)	Herpes zoster vaccine	1 (2.4)
Europe (not specified)	1 (2.4)	Human Papilloma Virus (HPV) vaccine	9 (21.4)
France	2 (4.8)	Hypothetical vaccine	5 (11.9)
Germany	3 (7.1)	Influenza vaccine	8 (19.0)
Hong Kong	3 (7.1)	Leptospirosis vaccine	1 (2.4)
Hungary	1 (2.4)	Meningococcal (B) vaccine	3 (7.1)
Italy	1 (2.4)	Pertussis vaccine	1 (2.4)
Japan	2 (4.8)	Pneumococcal vaccine	1 (2.4)
Netherlands, the	9 (21.4)	Rotavirus vaccine	2 (4.8)
Philippines, the	1 (2.4)	Tetanus-Diphtheria-Pertussis (Tdap) vaccine	1 (2.4)
Poland	2 (4.8)	Travel vaccines	1 (2.4)
South-Africa	1 (2.4)	Varicella vaccine	1 (2.4)
Spain	2 (4.8)		
Sweden	1 (2.4)		
Thailand	1 (2.4)		
United Kingdom	2 (4.8)		
Unites States of America	7 (16.7)		
Vietnam	1 (2.4)		

^a^As four studies included more than one country and one study covered multiple vaccines, the total number of studies exceeds the total amount of included studies (and 100%)

#### Choice task

The number of choice tasks ranged from four to 36, with most studies (40.5%) including less than ten choice tasks (Table 3). Ten studies (23.8%) used one method to identify appropriate attributes and levels, while 31 (73.8%) used more than one method listed in Table 3. Literature reviews and qualitative research such as focus groups were most popular (Table 3). Nearly all studies (92.9%) presented two or more vaccine scenarios per choice task and used a multinomial choice structure. Among these studies (*n* = 39), D-efficiency was most often used to pair and group choice profiles (35.9%). In addition, choice questions were mainly unforced and an option to remain undecided (opt-out) was provided (51.3%) (Table 3). Multiple descriptions were used to indicate the opt-out alternative (e.g. ‘no vaccination’, ‘neither’). Sixteen studies (41.1%) forced respondents to choose between two vaccine scenarios, the majority of them (*n* = 12) provided an opt-out in second instance (two-stage choice). Respondents were for instance asked if they would make the same choice in real life [34]. Two studies [35, 36] reported different formats in main texts and example questions.

**Table 3:** Overview of the design of the choice tasks among included studies

Aspect	Specification	Number of studies (%)
**Methods to identify attributes**^a^	Characteristics vaccine, disease	2 (4.8)
	Expert consultation	19 (45.2)
	Literature review	33 (78.6)
	Previous DCE	4 (9.5)
	Qualitative research	28 (66.7)
	Theories vaccine decision-making	1 (2.4)
	Vaccination policy	1 (2.4)
	Not reported	1 (2.4)
**Choice structure**	Binary	3 (7.1)
	Multinomial	39 (92.9)
**Methods to create choice sets, of multinomial studies (*n*** **= 39)**^a^	D-efficiency	3 (7.7)
	D-efficiency using software	11 (28.2)
	Fold-over	3 (7.7)
	Random	3 (7.7)
	Random using software	1 (2.6)
	Other software	5 (12.8)
	Not reported	13 (33.3)
**Format of choice question, of multinomial studies (*n*** **= 39)**^b^	Forced choice	4 (10.3)
	Forced choice, followed by opt-out	12 (30.8)
	Unforced choice with opt-out	18 (46.2)
	Unforced choice with opt-out, followed by forced choice	2 (5.1)
	Unclear	3 (7.7)
**Number of choice tasks**	< 10	17 (40.5)
	10–15	10 (23.8)
	> 15	10 (23.8)
	Not reported	5 (11.9)

^a^As multiple methods could be used to identify attributes, the total number of studies exceeds the total amount of included studies (and 100%); ^b^Note the proportion (%) is calculated from studies applying multinomial choice structures (*n* = 39), not from the total amount of studies (*n* = 42)

#### Experimental design

An overview of the experimental designs used across studies is presented in Table 4. A fractional factorial design was used in 35 studies (83.3%). Only one study (2.4%) used all possible combinations (full factorial design). Among the studies reporting their type of design (*n* = 36), a range of software packages was used, with Ngene being most popular (25.0%). Eight studies (22.2%) used approaches other than software such as a catalog or a manual approach. Studies that included interaction terms along with main effects (*n* = 23) generally used more choice tasks than the studies analysing main effects only (*n* = 2). Although seventeen studies (40.5%) did not provide details on their design plan in the main text, primary analyses of thirteen studies showed that it was restricted to main effects (Table 4).

**Table 4:** Overview of the experimental design and conduct of included studies

Aspect	Specification	Number of studies (%)^b^
**Type of design**	Fractional factorial design	35 (83.3)
	Full factorial design	1 (2.4)
	Not reported	6 (14.3)
**Design plan**	Main effects	2 (4.8)
	Main and interaction effects	23 (54.8)
	Not reported, main effects in primary analysis	13 (31.0)
	Not reported, main & interaction effects in primary analysis	1 (2.4)
	Not reported, unclear in analysis	3 (7.1)
**Software/approach, of studies reporting type of design (*n*** **= 36)**^a^	Ngene	9 (25.0)
	SAS	3 (8.3)
	Sawtooth	3 (8.3)
	SPSS	2 (5.6)
	Other computer algorithm	3 (8.3)
	Catalog approach	5 (13.9)
	Manual	1 (2.8)
	Other approach	2 (5.6)
	Not reported	8 (22.2)
**Piloting**	Yes	34 (81.0)
	No	1 (2.4)
	Not reported	7 (16.7)
**Mode of administration**	Interview-administered	5 (11.9)
	Self-administered	34 (81.0)
	Both	1 (2.4)
	Not reported	2 (4.8)
**Sample size**	< 200	1 (2.4)
	200–400	14 (33.3)
	400–600	12 (28.6)
	600–800	6 (14.3)
	800–1000	2 (4.8)
	1000–1200	1 (2.4)
	≥ 1200	6 (14.3)
(**Financial) compensation**	Yes	15 (35.7)
	No	2 (4.8)
	Not reported	25 (59.5)

^a^Note that the proportion of the studies using particular software packages or approaches is taken from the studies reporting their type of design (*n* = 36) instead of all includes studies (*n* = 42); ^b^Due to rounding of percentages, the total may not count up to 100%

In total 226 vaccine attributes were included in the 42 choice experiments. The number of attributes per study ranged from three to eight, the number of levels per attribute ranged from two to seven. With regard to the overarching categories, 38.9% of the attributes were categorized as outcome, 24.8% as process, 23.0% as other and 13.3% as cost. Overall, eighteen domains were identified (3 outcome, 8 process, 1 cost, 7 other). Details on the categories and domains are delineated in the data comparison sections (Tables 7 and 8) and in Additional file 3.

#### Conduct

More than 80% of the studies reported a pilot and/or soft launch (Table 4). The size differed from four [37] to three hundred respondents [38] and from a single-stage [35] to multiple-stage procedures (e.g. combination of pre-pilot, pilot and soft launches) [39, 40]. The majority of self-administered surveys was completed online (25 studies). Sample sizes ranged from fifty [41] to 2505 respondents [42]. Most studies included between two and four hundred respondents (Table 4). Larger sample sizes were not necessarily accompanied by the use of stricter thresholds (e.g. *p* < 0.001). The rule of thumb proposed by Orme [43] was most often used to justify sample sizes of CAs. Half of the studies did not justify their sample size nor included sample size calculations (Table 4). A third of the studies (35.7%) compensated respondents in cash, vouchers or a physical gift, the value varied from £1–2 to $55 (Table 4).

#### Analysis

A summary of the approaches used to analyse data is presented in Table 5. Half of the studies applied mixed or random parameter logit models (MXL/RPL). Random or mixed effects logit models were most often used to analyse forced choices. Nearly all studies (97.6%) accounted for variation in preferences across groups. Subgroup analyses were either performed by using separate models for different groups or by incorporating interaction terms into the model. The methods used to distinguish subgroups are outlined in Table 5. Methodological aims drove the subgroup analyses of 29 studies. These studies used for instance different cost ranges for subgroups or compared groups who passed and failed the consistency/dominance tests and groups with and without preference to opt-out. With regard to the outcome measures, welfare measures such as WTP were most frequently used (45.2%), followed by probability or uptake analyses (42.9%). Least reported measures were market simulations, willingness-to-accept and positive or predictive value (see ‘other measures’ Table 5). Most studies (83.3%) used software to analyse the data. These packages were not necessarily the same as the ones used to construct experimental designs (e.g. Ngene vs. Nlogit in Hofman et al. [44]).

**Table 5:** Overview of approaches used to analyse data, the journal and source of funding

Aspect	Specification	Number of studies (%)^a^
**Econometric model Subgroup analysis**	Multinomial logit	12 (28.6)
	Generalized linear random effects logit	1 (2.4)
	Hierarchical Bayes	6 (14.3)
	Latent class	4 (9.5)
	Random effects logit	6 (14.3)
	Mixed logit (random parameter)	21 (50.0)
	Other	5 (11.9)
	Methodology related	29 (69.0)
	Previous experiences	5 (11.9)
	Sociodemographic factor(s)	32 (76.2)
	Vaccine beliefs/perception/knowledge	13 (31.0)
	Vaccine intention or behaviour	6 (14.3)
	Vaccination or health status	5 (11.9)
	Other	5 (11.9)
**Outcome measure**	Individual utility scores	3 (7.1)
	Odds ratio, change in log-odds	8 (19.0)
	Relative attribute importance	10 (23.8)
	Marginal rate of substitution (trade-off)	8 (19.0)
	Vaccine uptake/probability analysis	18 (42.9)
	WTP	19 (45.2)
	Other	4 (9.5)
**Analysis software**	JMP Pro	2 (4.8)
	Nlogit	9 (21.4)
	SAS	11 (26.2)
	Sawtooth	4 (9.5)
	SPSS	3 (7.1)
	Stata	11 (26.2)
	Other	10 (23.8)
	Not reported	7 (16.7)
**Journal**	Clinical	25 (59.5)
	Economic	6 (14.3)
	General	4 (9.5)
	Marketing	1 (2.4)
	Methodological	1 (2.4)
	Pharmaceutical	1 (2.4)
**Funding**^b^	Yes Industry-funded	11 (28.2)
	Non-industry-funded	27 (69.2)
	No	1 (2.6)

^a^Totals exceed the total number of studies included in this review, since 13/42 studies used more than one econometric model, 28/42 used more than one approach to identify subgroups, 23/42 used more than one outcome measure, 10/42 used more than one software package; ^b^Note, the source of funding is based on the studies reporting their source of funding (*n* = 39) instead of all includes studies (*n* = 42)

#### Journal & funding

The majority of the studies (59.5%) were published in clinical journals (Table 5). Of the 39 studies reporting their source of funding, approximately a quarter was funded or supported by a pharmaceutical or manufacturing company producing the vaccine under study (*n* = 11). The remaining studies, except for Ngorsuraches et al. [45], received a research grant of governmental bodies, non-profit organizations or research/education institutes (Table 5).

### Quality assessment

An overview of the quality scores of all 42 studies is presented in Table 6. Quality scores ranged from 5.5 to 12.5, with an average score of 9.3. Scores did not improve over time, since average scores of studies published between 2000 and 5, 2006–10, 2011–5 and 2016–20 were 8.8, 10.5, 9.1 and 9.4 respectively. However, industry-funded studies scored lower than non-industry funded studies (mean of resp. 8.5 and 9.5). Among the four categories distinguished in Table 6, studies scored best on *analysis* (mean: 0.84), followed by *choice task design* (mean: 0.70), *conduct* (mean: 0.65) and *experimental design* (mean: 0.55).

**Table 6:** Overview of the quality assessment of the included studies

**Criteria**	**Study**																					
	**Adams et al.** [39]	**Arbiol et al.** [46]	**Bishai et al.** [47]	**Brown et al.** [35]	**Brown et al.** [36]	**de Bekker-Grob et al.** [48]	**de Bekker-Grob et al.** [38]	**Determann et al.** [49]	**Determann** **et al.** [50]		**Eilers et al.** [51]	**Flood et al.** [52]	**Flood et al.** [53]	**Gidengil** **et al.** [54]		**Guo et al.** [55]	**Hall et al.** [41]	**Hofman et al.** [56]	**Hofman et al.** [44]	**Huang et al.** [57]	**Lambooij et al.** [58]	**Ledent et al.** [59]
Choice task design																						
Attributes and levels grounded in qualitative work with target population	1	0	0	1	1	0.5	1	1	1		0.5	1	1	0.5		0	0	1	0	1	1	0.5
No conceptual overlap between attributes	0	1	1	0.5	0.5	0.5	0.5	1	1		0.5	0.5	0.5	0.5		1	0.5	1	1	0.5	0.5	0.5
Uni-dimensional attributes	0.5	1	1	1	1	1	1	1	1		0.5	1	1	0.5		1	0.5	1	1	1	1	0.5
Opt-out/status quo option or justification forced choice	1	1	1	0.5	0.5	1	1	1	1		1	0	0	1		1	1	1	1	0	1	1
Experimental design																						
Experimental design optimal or statistically efficient	1	0.5	0.5	1	1	1	1	1	1		0	0	0	0		0.5	0.5	1	1	0	0	0.5
Conduct																						
Piloting conducted amongst target population	1	0.5	0	1	1	0.5	1	0.5	0.5		1	0.5	0	0.5		0	0	0.5	0.5	0	0	0.5
Target population(s) appropriate for research objective	1	1	1	1	1	1	1	1	1		1	1	1	1		1	1	1	1	0.5	1	1
Sampling frame representative of target population	0.5	0.5	0.5	1	0.5	1	1	1	1		0.5	0.5	1	0.5		0.5	0	1	1	1	1	0.5
Response rate sufficient to minimize response bias	0	0	0	0.5	0.5	1	1	0.5	0.5		0.5	0.5	0.5	0.5		1	1	0.5	1	0.5	0.5	0
Analysis																						
Any pooled analysis from different subgroups appropriate	1	1	1	0.5	1	1	1	1	1		1	0	0	1		0.5	1	0.5	1	0.5	0	1
Econometric model appropriate for choice task design	1	1	1	1	1	1	1	1	1		1	0.5	0.5	0.5		1	1	1	1	0.5	1	0.5
Econometric model accounts for serial correlation of choices	1	1	1	1	1	1	1	1	1		1	0	0	1		1	1	1	1	0	1	0
Relative attribute effects compared using a common metric	1	1	1	1	1	1	1	1	1		0	0	0	1		1	1	1	1	1	1	1
**Total validity score**	**10**	**9.5**	**9**	**11**	**11**	**11.5**	**12.5**	**12**	**12**		**8.5**	**5.5**	**5.5**	**8.5**		**9.5**	**8.5**	**11.5**	**11.5**	**6.5**	**9**	**7.5**
**Validity criteria**	**Study**																					
	**Liao et al.** [60]	**Liao et al.** [61]	**Lloyd et al.** [37]	**Marshall et al.** [42]	**Ngorsuraches et al.** [45]	**Oteng et al.** [62]	**Pereira et al.** [63]	**Poulos et al.** [64]	**Poulos et al.** [65]	**Poulos et al.** [66]	**Poulos et al.** [40]	**Sadique et al.** [67]	**Sapède et al.** [68]	**Seanehia et al.** [69]	**Shono et al.** [70]	**Shono et al.** [71]	**Sun et al.** [72]	**Veldwijk et al.** [34]	**Verelst et al.** [73]	**Verelst et al.** [74]	**Wang et al.** [75]	**Wong et al.** [76]
Choice task design																						
Attributes and levels grounded in qualitative work with target population	0	1	1	0	0	0.5	0.5	0	0	1	0.5	0	1	0	0	0	1	1	0	0.5	0	0
No conceptual overlap between attributes	0.5	0	0.5	1	0.5	0.5	0.5	0.5	0.5	1	1	0	1	1	1	1	0.5	1	0.5	0.5	1	1
Uni-dimensional attributes	1	1	1	0.5	1	1	1	1	0.5	0.5	1	0	1	1	1	1	1	1	0	0.5	0.5	1
Opt-out/status quo option or justification forced choice	1	1	0	1	1	1	0	1	1	1	1	1	1	1	1	1	0	1	0.5	0	0.5	1
Experimental design																						
Experimental design optimal or statistically efficient	0.5	0.5	0.5	0.5	0.5	0.5	0.5	1	1	0	0	0	0	0.5	0.5	0.5	0	1	1	1	1	0.5
Conduct																						
Piloting conducted amongst target population	0.5	0.5	1	1	0.5	0.5	0	1	1	1	0.5	1	0.5	1	0.5	0.5	0	1	0.5	1	1	1
Target population(s) appropriate for research objective	0.5	0.5	1	1	1	0.5	1	1	1	1	1	1	0.5	1	1	1	0.5	1	1	1	1	0.5
Sampling frame representative of target population	0.5	0	0	1	0.5	1	1	1	1	1	0.5	1	0	0.5	1	1	1	1	0.5	1	0.5	0.5
Response rate sufficient to minimize response bias	0	0	0	0	1	0	0	0.5	0	0.5	0	0	0	0	0	0	1	0.5	0	1	0	1
Analysis																						
Any pooled analysis from different subgroups appropriate	1	1	1	1	1	1	1	1	1	1	1	0	1	0.5	1	1	0.5	1	1	1	0.5	1
Econometric model appropriate for choice task design	1	1	0	1	0.5	1	0.5	1	1	1	0.5	1	1	1	1	1	0.5	1	1	1	1	1
Econometric model accounts for serial correlation of choices	1	1	0	1	0	1	1	1	1	1	0	1	1	1	0	1	1	1	1	1	1	0
Relative attribute effects compared using a common metric	0.5	0.5	1	1	0.5	1	1	1	1	1	1	1	1	1	1	1	1	1	1	1	0.5	1
**Total validity score**	**8**	**8**	**7**	**10**	**8**	**9.5**	**8**	**11**	**10**	**11**	**8**	**7**	**9**	**9.5**	**9**	**10**	**8**	**12.5**	**8**	**10.5**	**8.5**	**9.5**

With respect to the category *choice task design*, the majority of the studies used unidimensional attributes and included an opt-out in first or second instance (resp. 71.4 and 73.8%). Weaknesses were particularly observed in the identification of attributes/levels and in the occurrence of conceptual overlap between attributes. The second category, *experimental design*, was comprised of one criterion. The majority of the studies (64.3%) used (fractional) factorial designs that were sub-optimal (i.e. scored below 1). Furthermore, varying scores were administered on the criteria of the *conduct* category. While studies commonly tested survey features in a pilot and identified appropriate target populations, three-quarter reported response rates below 50% and almost half used inappropriate sampling frames. Almost all studies satisfied at least three of the four criteria incorporated in the last category *analysis*, particularly the ones concerning the economic model and use of a common comparable scale (metric) to interpret relative attribute effects [77, 78]. Some improvements could still be made in analysis of preferences of heterogenous populations, as pooled data might cover up preference differences between subgroups [30, 77].

When combining scores on the four categories into an overall score, sixteen of the 42 studies (38.1%) had a total score of at least 10 and passed the quality assessment (Fig. 1). These were regarded as ‘high-quality studies’ and were included in the data comparison. Total scores of the remaining studies (61.9%) were insufficient to exclude most threats to validity (score < 10). These ‘lower-quality studies’ were hence only included in the robustness analyses. A more detailed description of the quality assessment is enclosed in Additional file 4.

### Comparison of high-quality studies

Of the sixteen high-quality studies, seven focused on vaccinees (43.8%), six on representatives (37.5%) and one on health advisors (6.3%). As it is difficult to draw firm conclusions on a single study, the study on health advisors [65] was added to the representatives’ category. Two studies [42, 74] addressed vaccinees as well as representatives (12.5%). As both reported preferences for vaccinees and representatives separately (per class), classes covering vaccinees were incorporated into the analysis of vaccinees and classes covering representatives into the analysis of representatives. Therefore, data of nine studies was compared for both target groups. Information on vaccine attributes of high-quality studies is summarized in Additional file 5.

#### Vaccinees

**Fig. 2:**
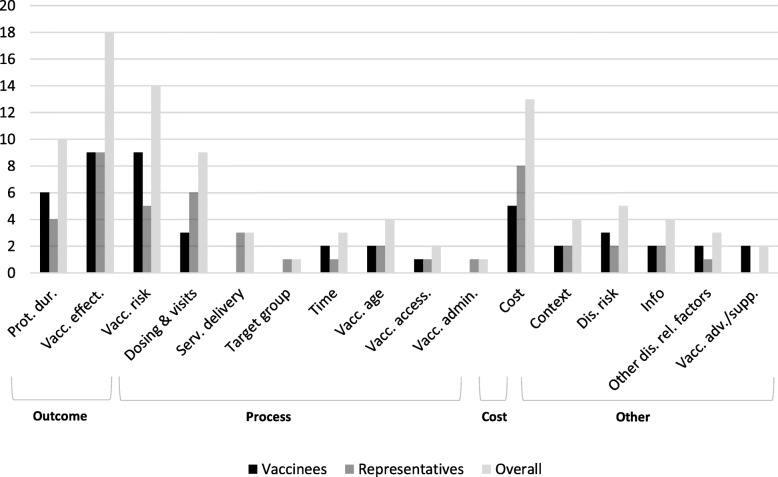
Frequency of domains in high-quality studies

Studies capturing preferences of vaccinees used 48 attributes, of which 50% were classified as outcome, 16.7% as process, 10.4% as cost and the remaining 22.9% as other. Thirteen domains were identified in total (three outcome, four process, one cost and five other). Figure 2 presents the total amount of attributes incorporated in each category and domain. Importance rankings derived from this figure are outlined in Additional file 6. Most frequently used outcome measures were vaccine effectiveness and vaccine risk (both 18.8%), followed by protection duration (12.5%) and cost (10.4%). Vaccine effectiveness referred to the level of protection that a vaccine provided against a disease or to the deaths/illnesses prevented over a certain time span. Vaccine risk referred to the frequency of (mild or serious) side effects after vaccination. Context, information, other disease related factors, vaccine advice/support were also reported, but could not be grouped in any of the three categories. They are classified as other. ‘Context’ referred to vaccine coverage rates at local and population level, ‘information’ to the media coverage or attention about the vaccine and ‘vaccine advice/support’ to recommendations of family, friends, doctors, governmental bodies and international organizations. Process-related domains were least reported. Vaccine accessibility was for instance only included in the study of Verelst, Willem, Kessels & Beutels (2.1%) [74]. Statistical significance was reported in all nine studies. The average sample size was 1113 and three *p*-value thresholds were used to determine if attributes were statistically significant (Table 7). Vaccine risk and vaccine effectiveness, both outcome measures, were most often statistically significant (resp. 15 and 14 times). However, the latter was not found to be significant for one class of the study of Determann et al. [50]. Domains that were also commonly statistically significant included: cost, protection duration, dosing & visits, information and vaccine advice or support (Table 7). The importance of the former three was also confirmed in the frequency of reporting (Fig. 2). Factors other than disease risk, such as the spread of the disease and availability of curative treatments, were grouped under the domain ‘other disease related factors’ (other). This domain as well as the domain ‘time’ (process) were not statistically significant in any of the studies (Table 7).

**Table 7:** Overview of high-quality studies reporting relative statistical significance (vaccinees)

Category & domain (*n*=)*	Statistical significance**				
	***P*** < 0.10	***P*** < 0.05	***P*** < 0.01	Total	Not significant
**Outcome**					
Protection duration (*n* = 6)	0	3	5	8	1
Vaccine effectiveness (*n* = 8)	0	5	9	14	1
Vaccine risk (*n* = 7)	0	4	11	15	0
**Process**					
Dosing & visits (*n* = 3)	1	1	3	5	1
Time (*n* = 2)	0	0	0	0	2
Vaccination age (*n* = 2)	0	1	2	2	1
Vaccine accessibility (*n* = 1)	0	1	0	1	0
**Cost**					
Cost (*n* = 5)	0	1	8	9	0
**Other**					
Context (*n* = 1)	0	2	0	2	0
Disease risk (*n* = 2)	0	1	2	3	0
Information (*n* = 2)	1	1	3	5	0
Other disease related factors (*n* = 1)	0	0	0	0	2
Vaccine advice/support (*n* = 2)	1	0	4	5	0

*n = number of studies reporting domains. All nine studies reported statistical significance; ** Information is based on main models and pooled data when available (if not, data of separate models/classes wasused). Some studies included more than one attribute related to a particular domain. Totals could hence exceed the total number of studies incorporated.

#### Representatives

In total, 48 attributes were identified in nine studies capturing vaccine preferences of representatives (Fig. 2). Of these attributes, 37.5% were outcome-related, 31.3% were process-related and 16.7% were cost-related. The remaining 11.6% of the attributes were categorized as other. Overall, fifteen domains were identified (three outcome, seven process, one cost, four other). In contrast to vaccinees, attributes regarding vaccine advice or support were not reported. However, three process-related domains were added: service delivery, target group and vaccine administration. ‘Service delivery’ covered practical aspects such as vaccine location and availability of appointments, while ‘vaccine administration’ referred to the mode of administration (e.g. injection). Vaccine effectiveness was most frequently reported (18.8%), followed by cost (16.7%), dosing & visits (12.5%), vaccine risk (10.4%) and protection duration (8.3%). In line with studies targeting vaccinees, vaccine accessibility was least reported. Two studies [65, 66] did not report statistical significance or did not provide a legend (description). For the study of Shono & Kondo [71], statistical significance could be determined based on reported *p*-values. Therefore, seven studies were included in Table 8. On average 1037 respondents were included and among the four thresholds used, *p* < 0.05 was most commonly applied. All attributes included in high-quality studies were statistically significant in one or more study/studies (Table 8). In line with the frequency of reporting, vaccine effectiveness and cost were most commonly statistically significant, followed by vaccine risk and protection duration. Domains that were least used were also least reported statistically significant (Table 8). These were categorized as process and other. Varying results were reported for information (other) and service delivery (process) (Table 8). The professional administering vaccines was for instance statistically significant, while the availability of appointments and location were not [39]. Information was found to be statistically significant half of the times, as information on benefits and risks were statistically significant, but the format was not [39].

**Table 8:** Overview of high-quality studies reporting relative statistical significance (representatives)

Category & domain (*n*=)*	Statistical significance**					
	P < 0.10	P < 0.05	P < 0.01	P < 0.001	Total	Not significant
**Outcome**						
Protection duration (*n* = 4)	0	1	2	1	4	1
Vaccine effectiveness (*n* = 6)	0	3	3	1	7	0
Vaccine risk (*n* = 5)	1	4	0	0	5	0
**Process**						
Dosing & visits (*n* = 2)	0	1	1	0	2	0
Service delivery (*n* = 2)	0	1	0	0	1	2
Target group (*n* = 1)	0	0	1	0	1	0
Time (*n* = 1)	0	0	1	0	1	0
Vaccination age (*n* = 1)	0	1	0	0	1	0
Vaccine accessibility (*n* = 1)	0	1	0	0	1	0
**Cost**						
Cost (*n* = 5)	0	1	4	1	6	0
**Other**						
Context (*n* = 1)	0	2	0	0	2	0
Disease risk (*n* = 1)	0	1	0	0	1	0
Information (*n* = 1)	0	0	1	0	1	1

*n = number of studies reporting domains. 7/9 studies reported statistical significance (incl. legend) and/or *p*-values; **Information is based on main models and pooled data when available (if not, data of separate models/classes was used). Some studies included more than one attribute related to a particular domain. Totals could hence exceed the total number of studies incorporated.

#### Overall preferences and comparison of vaccinees and representatives

A total of 96 attributes were identified in high-quality studies. Attributes were most commonly classified as outcome (43.8%), followed by process (24.0%), other (18.8%) and cost (13.5%). Attributes covered sixteen domains. Figure 2 showed that the same domains were valued by vaccinees and representatives. The outcome measure vaccine effectiveness was most often preferred regardless target group. The order of the remaining domains showed slight differences (Additional file 6). Vaccine risk as well as duration of protection (both outcome-related), were for instance more important for vaccinees compared to representatives, while representatives valued costs of vaccines and dosing & visits more than vaccinees (cost and process-related). For both target groups, vaccine accessibility (process) was least preferred. Statistical significance was reported (or could be determined) in sixteen studies and was most often defined by using *p* < 0.01. The average sample size was 1080. Studies with sample sizes above 500 usually applied multiple thresholds to determine at which point attributes were (not) statistically significant (e.g. p < 0.01, *p* < 0.05 and *p* < 0.10). Although studies capturing preferences of vaccinees reported statistical significance more often, overall results showed that for both target groups outcome and cost-related domains were most frequently significant. The domain other disease related factors (other) was not statistically significant and vaccine accessibility (process) was an equal amount of times significant (at p < 0.05) and insignificant (Additional file 7).

### Comparison of high- and lower-quality studies

Eight lower-quality studies focused on vaccinees, fifteen on representatives and three on health advisors (Additional file 5). In line with the approaches used for high-quality studies, studies targeting health advisors [37, 61, 70] were added to the representatives’ category and outcomes of Verelst, Kessels, Delva, Beutels & Willem [73] were split (and grouped under vaccinees as well as representatives). As a result, eighteen studies targeted vaccinees and 27 representatives.

A total of 243 attributes were used, most of which were outcome-related (39.1%). Compared to high-quality studies, two domains were introduced by lower-quality studies: vaccine content and other (both other). The former referred to substances/components of vaccines such as preservatives and the latter included attributes that could not be grouped under the other seventeen domains (e.g. vaccine testing). All domains that were most often reported and most commonly statistically significant corresponded with high-quality studies, except for disease risk (other). An increased preference for disease risk was observed across both target groups. Another inconsistency refers to the sample sizes. Average sample sizes of lower-quality studies were 518 compared to 1080 in high-quality studies. Lower-quality studies tended to use stricter *p*-value thresholds for sample sizes below 500, while *p* < 0.05 was often used for sample sizes above 500. In accordance with high-quality studies, domains related to process and other were least found statistically significant for both target groups. A more detailed comparison of high- and lower-quality studies in enclosed in Additional file 8.

## Discussion

The growing body of SP literature on vaccination highlights the increased interest in the use of choice-based experiments, to elicit preferences for a variety of vaccines and to understand factors influencing vaccine decision-making of different groups of individuals. A total of 42 studies were identified in this review, capturing preferences of three different target groups and covering fourteen vaccines or vaccine programs. Given the limited amount of studies assessing preferences of health advisors, this review focused on examining and comparing preferences for vaccine attributes of vaccinees and representatives (including health advisors). The former generally focused on preferences of adults and adolescents, while the latter mainly captured parental preferences for childhood vaccines.

Among the 42 included studies, sixteen studies were of high-quality and could be included in the comparison of vaccine preferences. Irrespective of target group captured, outcome-related attributes, such as vaccine effectiveness, vaccine risk and protection duration, were most frequently reported, followed by attributes covering the monetary cost of vaccines. Outcome- and cost-related attributes were also most commonly statistically significant across all studies, indicating that the same factors are generally preferred across different groups of individuals. Correspondence was also observed for least preferred attributes, since attributes related to a vaccines’ access were least valued by both target groups. However, it should be noted that elements of accessibility might already be included in other attributes such as in cost (see Verelst et al. [73]). Therefore, it should be interpreted cautiously.

The overall finding is in line with the review of Lack et al. [21], which focused on HPV-vaccination and found that vaccinees, parents and providers have the strongest preferences for attributes related to vaccine outcomes. Comparable patterns were also identified among earlier reviews of CAs [20] and DCEs [19]. Michaels-Igbokwe et al. [19] indicated for instance that attributes related to degree/duration of protection and risk were most often statistically significant across DCEs studying preferences for childhood and adolescent vaccines. In addition, attributes included in DCEs generally addressed features of vaccines, while neglecting service (i.e. process) or contextual aspects (i.e. other). The latter was observed to a lesser extend in this review, as nearly half of the high-quality studies incorporated attributes describing coverage rates, waiting times, access, locations, information provision or social support. A more plausible explanation would be that aspects of a vaccine process are simply less important for vaccinees and representatives in making vaccine decisions. This hypothesis is supported by findings of Guo et al. [55], outlining that service convenience and quality are less ‘dramatic’ than vaccine features.

Current findings showed that outcome-related attributes were more often statistically significant in studies targeting vaccinees (esp. vaccine risk), while cost-related attributes were more often statistically significant in studies of representatives. This indicates that the level of evidence for outcomes and costs slightly differed between both target groups. However, outcome and cost parameters were statistically significant in both target groups, indicating no differences in preferences of vaccinees and representatives. Instead, differences for cost might be (partly) explained by the definition of this domain. Particularly among studies targeting representatives, cost-related attributes were operationalized differently (e.g. ‘type and value of parental reward’ and ‘payment for one doctor visit’). This might have affected the way in which respondents interpreted attributes and eventually how they valued vaccine scenarios.

The robustness analysis confirmed findings of the main analysis and only showed a slight increase in preference for attributes covering disease risk (in both target groups). This suggests, in line with qualitative research on vaccine behaviour and the Health Belief Model [79–81], that epidemiological and affective factors, such as the susceptibility to and severity of diseases, may also affect vaccine decisions. The discrepancy could also be caused by the conceptual overlap identified in the quality assessment. Four lower-quality studies included more than one risk-related attribute, while no high-quality study did. According to Mandeville et al. [30] overlap could distort parameter estimates, as attributes (and effects) are not distinct and do not vary independently. Respondents might for instance experience difficulties in distinguishing and interpreting attributes.

When examining characteristics of the studies, it is observed that all high-quality studies were conducted in HICs and applied MXL/RPL or LCM. They mainly focused on vaccines against sexually transmitted infections, while lower-quality studies were characterized by a broader range of vaccines, countries and econometric models. High-quality studies were also more likely to express outcomes in WTP or predicted vaccine uptake. The latter is in contrast to Clark et al. [17] who focused on general health preferences and observed a decline in the use of monetary values and probabilities. However, probabilities are particularly useful in vaccination, as herd immunity is an important externality which can only be acquired when vaccination coverage passes a certain threshold [14, 82, 83]. Adult and traveller vaccines might also require (co-)payments, which can be adequately captured in monetary values [77]. The trend of using more sophisticated designs and appropriate software, observed in the review of Soekhai [18], is reinforced by current findings. A last observation was that high-quality studies used larger sample sizes compared to lower-quality studies. Moreover, the high-quality studies with larger sample sizes (≥500) were inclined to use multiple thresholds (i.e. alphas), whereas lower-quality studies used smaller alphas for sample sizes below 500 and *p* < 0.05 for sample sizes above 500. Which is contradicting with previous research that indicates that larger sample sizes are required when lowering alpha and vice versa [84, 85].

In the quality assessment, the average score was almost one point higher than reported by Michaels-Igbokwe et al. in 2017 (8.4 vs. 9.3) [19]. This suggests that choice-based experiments improved elements of design. However, no improvement was observed in our quality scores per period. The quality assessment also indicated that industry-funded studies scored remarkably lower than non-industry funded studies. This addresses the need to get insight into industry sponsorship and used methodology. In line with previous reviews who used the 13-criteria checklist [19, 30], no study reached the maximum score: all failed at least one criterion. Weaknesses were particularly observed on elements of choice task design, experimental design and conduct. This underlines once again the technical requirements for all four stages and highlights the need to improve scientific rigour across choice-experiments in health.

### Strengths and weaknesses

A strength of this study is the use of a formal quality assessment tool [30] to critically appraise the methodological quality and internal validity of included studies. Due to this tool and the quality threshold, conclusions regarding the drivers of vaccine decisions were based on attributes of high-quality studies only [30, 32, 86]. The robustness was also tested and confirmed. Based on this, it could be ascertained that findings were largely not affected by exclusion of lower-quality studies [87]. The comprehensiveness of the search is also a strength. The primary search was updated and related reviews were screened. Only two additional studies were identified, confirming the accuracy of key words used and suggesting that the primary was all-encompassing [26]. However, data was extracted from published literature and relied on what was reported in articles and available supplementary material. Like in any review, reporting and publication bias could hence not be eliminated [86, 88]. In contrast to previous research, no in- or exclusion criteria were formulated based on vaccine topic or site examined. Included studies covered a variety of vaccines/programs, populations and settings, which promoted the generalisability of results [87]. Due to the limited research on preferences in low-resource countries and of healthcare professionals, both were still underrepresented which may hamper the generalisability to these particular populations and settings.

Beside strengths, some limitations could also be identified. Key steps of this review were for instance performed by a single researcher, which may have induced reporting bias [32, 86, 88]. To minimize the occurrence of inconsistencies/mistakes, all steps were closely monitored and checked by a second researcher and ambiguities were discussed and agreed upon. To reduce the number of attributes, the commonly used classification of outcome, cost and process was used [21, 89–91]. However, multiple attributes could not be classified properly, and a fourth category needed to be added. The variety of attributes included hampered appropriate naming and interpretation of this category as a whole. Besides, we decided to include health advisors into the representatives’ group, because both referred to individuals that make vaccine decisions for others. Only four studies focused on health advisors, due to which it was considered inappropriate to create a separate group. Analyses revealed that no new domains were introduced by the studies of health advisors, indicating that this decision had no influence on the findings about representatives. Last of all, drivers were based on frequency of reporting and statistical significance of domains instead of relative importance scores per attribute. Given the range of vaccines, attributes, choice tasks, populations and outcome measures within included studies, a meta-analysis was not possible [92]. Although both measures give an indication about the importance of attributes, the adequacy is discussible. Statistical significance is not only contingent upon the set of attributes used, but also on the way in which it is defined [32, 84, 85]. Different choices in p levels (for instance *p* < 0.10 vs *p* < 0.01) can influence the frequencies reported per domain. Moreover, the frequency of reporting domains was also skewed by studies including more than one attribute of the same domain. Both hampered interpretability and may have induced bias on outcome level. Nonetheless, this was tried to minimize by accounting for significance levels (alphas) used and the amount of studies reporting certain domains.

### Implications for research and policy

#### Implications for research

The quality assessment showed that the choice task, experimental design and elements of conduct received less attention compared to analysis. Studies conducted in LMICs particularly reported inappropriate experimental designs, showed conceptual overlap and failed to pilot test the survey. Although Michaels-Igbokwe et al. [19] observed similar methodological patterns in 2017, choice experiments in vaccination have not yet structurally improved their designs and conduct. The time lag between the conduct and publication of results could play a role, as high-quality studies were on average published three years after its conduct. Improvements in choice design and conduct are notwithstanding crucial to ensure reliable estimates of vaccine preferences. As recommended by Soekhai et al. [18] this might be facilitated by formulation of guidelines to report choice experiments. Furthermore, future research could broaden the approaches used to measure SP (e.g. add contingent valuation) to adequately capture preferences of health professionals. Literature [9, 93, 94] showed that decision strategies particularly differ for medical professionals and vaccinees. In this review only a limited amount of studies on preferences of health professionals could be included. Additional research could also focus on target groups other than those distinguished in this review (e.g. policy makers, based on gender) or on vaccine preferences in low-resource settings. A combination would also be interesting as qualitative research [95, 96] indicated that national decision-makers in LMICs particularly preferred simplified delivery mechanisms, thermostability and an extended shelf-life. In light of the current corona pandemic, it would also be worthwhile to assess preferences for (future) vaccines against epidemic infections, such as COVID-19 and SARS.

#### Implications for policy

In contrast to previous qualitative studies [9, 11, 12], this review demonstrates that vaccine preferences show similar patterns for vaccinees and representatives. Broadly the same strategies could be adopted to promote and optimize vaccination behaviour. Strategies should focus on outcomes, for instance by providing proper and understandable information about the effectiveness of vaccines, duration of effectiveness and risks associated with vaccine administration, dosing and handling. Insight into the latter is particularly important for vaccinees. Effective pricing strategies should be applied (if applicable) when introducing or continuing the use of vaccines. Particularly for vaccine decisions that involve representatives (e.g. childhood vaccines), the element of cost is important. The robustness analysis indicates that disease risk is important for vaccinees and representatives as well. Therefore, information strategies should not only cover vaccine-related aspects, but should also inform target groups about the severity and probability of diseases. Across included studies, individuals value a reduced number of doses/visits when deciding for themselves and others. Vaccine programs that currently include multiple injections (such as HPV and COVID-19 vaccines), should hence try to minimize the amount of dosages as much as possible.

## Conclusion

Where previous literature reviews were restricted to specific target groups, type of vaccines or formats of choice experiments, this review was the first to examine vaccine preferences of different target groups across vaccines. A clear and comprehensive overview of current SP literature was provided, which did not only give insight into the four main drivers of vaccine decision-making and the correspondence between vaccinees and representatives, but also indicated room for improvement across three of the four stages of choice experiments. Future research into vaccine preferences of target groups other than vaccines and representatives and among groups in low-resource settings would give insight into the generalizability of current findings.

## Supplementary Information

**Additional file 1:** Overview of search strategy. A table describing the search applied in each of the databases.

**Additional file 2:** Overview of study characteristics. A complete overview of the characteristics of all studies included in this review.

**Additional file 3:** Overview of attributes included in each category/domain. A table giving insight into the way attributes were grouped under categories and domains.

**Additional file 4:** Quality assessment. A detailed description of the outcomes of the quality assessment performed.

**Additional file 5:** Overview of vaccine attributes per study. A detailed overview of the vaccine attributes and domains used in each of the studies (incl. Statistical significance).

**Additional file 6:** Importance rankings vaccine attributes. Tables outlining the frequency in which domains were reported among high- and lower-quality studies.

**Additional file 7:** Relative statistical significance (overall preferences). Tables giving insight into the amount of times domains were reported statistically significant in all studies (overall preferences).

**Additional file 8:** Comparison of high- and lower-quality studies. A more detailed description of findings of the robustness analysis.

## Data Availability

The datasets used and/or analyzed during the current study are available from the corresponding author on reasonable request.

## Abbreviations

ADCE: Adaptive discrete choice experiment
CA: Conjoint analysis
CLM: Conditional logit model
DCE: Discrete choice experiment
HIC: High-income-country
ISPOR: International society for pharmacoeconomics and outcomes research
LCM: Latent class model
LMIC: Low-middle-income-country
MNL: Multinomial logit model
MXL: Mixed logit model
RP: Revealed preferences
RPL: Random parameter logit model
SP: Stated preferences
UMIC: Upper-middle-income country
WTP: Willingness-to-pay
