# Supplementary material for: Vaccine preferences driving vaccine-decision making of different target groups: a systematic review of choice-based experiments
Source: BMC Infect Dis. 2021 Aug 28;21:879. doi: 10.1186/s12879-021-06398-9 (PMC8397865; doi:10.1186/s12879-021-06398-9)
Supplement: Supplementary file 1 — Additional file 1: Overview of search strategy. A table describing the search applied in each of the databases. [file 12879_2021_6398_MOESM1_ESM.docx]

**Title page**

**Vaccine preferences driving vaccine-decision making of different target groups: A systematic review of choice-based experiments**

Marilyn Emma Diks (first author)

Faculty of Health Medicine and Life sciences, Maastricht University

Universiteitssingel 40, 6229 Maastricht, Netherlands

[marilyndiks@gmail.com](mailto:marilyndiks@gmail.com)

Mickael Hiligsmann

Department of Health Services Research, Care and Public Health Research Institute (CAPHRI), Maastricht University

Duboisdomein 30, 6229 Maastricht, Netherlands

[m.hiligsmann@maastrichtuniversity.nl](mailto:m.hiligsmann@maastrichtuniversity.nl)

Ingeborg Maria van der Putten (last and corresponding author)

Department of Health Services Research, Care and Public Health Research Institute (CAPHRI), Maastricht University

Duboisdomein 30, 6229 Maastricht, Netherlands

[i.vanderputten@maastrichtuniversity.nl](mailto:i.vanderputten@maastrichtuniversity.nl)

**Additional file 1 – Overview of search strategy**

An overview of the search as applied in the various databases, is presented in the table below. This table encompasses the articles that were initially identified through the database search (performed in April 2020) and, hence, still includes duplicates and articles that were considered ineligible after screening the title, abstract, full-text or methodological quality.

**Table 1 Search strategy in databases**

| **Database** | **Search terms** | **Filters used** | **Records identified (N= 546)** |
| --- | --- | --- | --- |
| CINAHL | ( vaccin* or immunis* or immuniz* or MH “Vaccines+” or MH “Immunization+”) AND (discrete choice or stated preference or DCE or choice experiment or conjoint analysis ) AND ( preference or MH “Patient preference+”) | **Limiters** - English Language  **Expanders** - Apply equivalent subjects  **Search modes** - Boolean/Phrase | 24 |
| EconLit | (vaccin* or immunis* or immuniz*) AND (discrete choice or stated preference or DCE or choice experiment or conjoint analysis) AND (preference) | **Expanders** - Apply equivalent subjects  **Narrow by Language:**- english  **Search modes** - Boolean/Phrase | 17 |
| Embase | ((vaccin* or immunis* or immuniz*).mp. or exp vaccine/ or exp vaccination/ or exp immunization/) and (discrete choice or stated preference or DCE or choice experiment or conjoint analysis).mp. and (preference.mp. or exp patient preference/) | limit to english language | 68 |
| PubMed | ((((((((((vaccin*) OR immunis*) OR immuniz*) OR vaccines[MeSH Terms]) OR vaccination[MeSH Terms]) OR immunization[MeSH Terms]) AND ((((discrete choice) OR stated preference) OR DCE) OR choice experiment OR conjoint analysis)))))) AND (((preference) OR patient preference[MeSH Terms])) | Filters: **Humans; English** | 52 |
| Web of Science | TS=(vaccin* OR immunis* OR immuniz*) AND TS=(discrete choice OR stated preference OR DCE OR choice experiment OR conjoint analysis) AND TS=(preference) | **Refined by:**  **LANGUAGES:** ( ENGLISH )  *Indexes=SCI-EXPANDED, SSCI, A&HCI, CPCI-S, CPCI-SSH, ESCI Timespan=All years* | 385 |
