# Supplementary material for: Vaccine preferences driving vaccine-decision making of different target groups: a systematic review of choice-based experiments
Source: BMC Infect Dis. 2021 Aug 28;21:879. doi: 10.1186/s12879-021-06398-9 (PMC8397865; doi:10.1186/s12879-021-06398-9)
Supplement: Supplementary file 2 — Additional file 2: Overview of study characteristics. A complete overview of the characteristics of all studies included in this review. [file 12879_2021_6398_MOESM2_ESM.docx]

**Additional file 2 – Overview of study characteristics**

The tables included in this file (1-5) summarize all characteristics of included studies (high- and lower-quality studies). Information on vaccine attributes is not included in this overview, but is outlined elsewhere (in Additional file 5).

**Table 1 Summary of the study characteristics of all included studies**

| Study | Study characteristics | | | | |
| --- | --- | --- | --- | --- | --- |
|  | **Year** | **Country** | **Vaccine type** | **Study population** | **Objective** |
| Adams et al. [1] | Publication: 2015  DCE: 2014-2015 | UK (HIC) | Childhood | Parents/caregivers of preschool children (<5 yrs)  Target group: representatives | Assess parental preferences for vaccination programs, policy recommendations on information of parental preferences for designing vaccination programs, predict vaccine uptake (under scenarios) |
| Arbiol et al. [2] | Publication: 2015  DCE: 2012 | PHL (LMIC) | Leptospirosis | Urban residents (adults)  Target group: vaccinees | Assess preferences for vaccine attributes, assess how demographic factors affect vaccine acceptance, estimate WTP for future vaccine |
| Bishai et al. [3] | Publication: 2007  CA: not reported | FR, DE (both HIC) | Meningococcal | Parents of teenage children  Target group: representatives | Assess effects of price, vaccine attributes and parents’ informational state on willingness to purchase of meningococcal vaccines out-of-pocket |
| Brown et al. [4] | Publication: 2010  CA: 2008 | USA (HIC) | HPV | Mothers of at least 1 daughter (13-17 yrs) who had not yet received HPV vaccine  Target group: representatives | Estimate relative importance of vaccine features, assess whether preferences differ by individual characteristics, estimate average maximum WTP, predict uptake under a variety of scenarios |
| Brown et al. [5] | Publication: 2014  CA: 2008 | USA (HIC) | HPV | Girls (13-17 yrs) who had not received HPV vaccine  Target group: vaccinees | Assess girls’ preferences for vaccine attributes, determine if preferences differ by individual characteristics and if small group has strong preferences against vaccination while the rest clearly differentiate between vaccine features, estimate vaccine uptake, estimate WTP |
| de Bekker-Grob  et al. [6] | Publication: 2010  DCE: not reported | NL(HIC) | HPV | Girls (12-16 yrs)  Target group: vaccinees | Investigate if girls make trade-offs between vaccine attributes, assess relative weights of vaccine attributes |
| de Bekker-Grob  et al. [7] | Publication: 2018  DCE: not reported | NL (HIC) | Influenza | General population (≥60 yrs)  Target group: vaccinees | Quantify how vaccination and patient characteristics impact influenza vaccination uptake of elderly, clinical recommendations for GPs to inform patients and policy recommendations for policy makers to tailor general brochures |
| Determann et al. [8] | Publication: 2014  DCE: 2013 | NL (HIC) | Hypothetical pandemic | General population  Target group: vaccinees | Investigate preferences of general population for pandemic vaccinations, calculate expected uptake, develop evidence-based behavioural and communication strategy for health professionals and agencies |

| Study | Study characteristics | | | | |
| --- | --- | --- | --- | --- | --- |
|  | **Year** | **Country** | **Vaccine type** | **Study population** | **Objective** |
| Determann et al. [9] | Publication: 2016  DCE: 2013 | NL, PL, SP, SE (all HIC) | Pandemic, influenza like (hypothetical) | General, adult population  Target group: vaccinees | Quantify and compare preferences of European citizens for vaccination programs for future pandemics, calculate expected uptake of vaccination under different pandemic scenarios, policy recommendations for preparedness plans and communication strategies |
| Eilers et al. [10] | Publication: 2017  DCE: 2014 | NL (HIC) | Pneumococcal  Herpes zoster  Pertussis  Influenza | Older adults (≥50 yrs)  Target group: vaccinees | Reveal relative importance of vaccine and disease attributes, final acceptance of four vaccines among Dutch older adults |
| Flood et al. [11] | Publication: 2011  CA: 2009 | USA (HIC) | Influenza | Children (8-12 yrs)  Target group: vaccinees | Examine children’s preferences for influenza vaccine attributes and factors influencing preferences |
| Flood et al. [12] | Publication: 2011  CA: 2009 | USA (HIC) | Influenza | Parents of children (2-12 yrs)  Target group: representatives | Examine parents’ preferences for vaccine attributes, identify key attributes and factors influencing preferences, determine if preferences vary based on parent’s likelihood of vaccinating their child |
| Gidengil et al. [13] | Publication: 2012  DCE: 2010 | USA (HIC) | Childhood | General, adult population  Target group: representatives | Measure parental and societal values and WTP for childhood combination vaccines |
| Guo et al. [14] | Publication: 2017  DCE: not reported | CHN (UMIC) | HepB | Adults (≥22 yrs)  Target group: vaccinees | Investigate adults’ preferences for convenience and quality of vaccination service, calculate private economic benefit from attributes, predict uptake rate for different vaccine scenarios |
| Hall et al. [15] | Publication: 2002  DCE: not reported | AU (HIC) | Varicella | Parents/guardians with at least one child (<12 yrs) who never had chickenpox  Target group: representatives | Predict uptake across range of hypothetical programs among parents |
| Hofman et al. [16] | Publication: 2014  DCE: 2009 | NL (HIC) | HPV | Parents of daughters (10-12 yrs)  Target group: representatives | Generate knowledge about potential improvements to HPV vaccination information and organization strategies, assess parental preference for vaccine attributes and uptake, assess trade-offs between attributes |
| Hofman et al. [17] | Publication: 2014  DCE: 2011 | NL (HIC) | HPV | Girls (11-15 yrs) who were (not) invited to get vaccinated  Target group: vaccinees | Assess which attributes influenced girls’ preferences for HPV vaccination uptake after media debates, comparison of DCEs, recommendations for information provision |
| Huang et al. [18] | Publication: 2020  DCE: 2017 | CHN(UMIC) | Childhood | Parents/caregivers of infants (<3 mos) at public immunization clinics  Target group: representatives | Assess parents’ preferences for vaccine program attributes, estimate WTP |
| Lambooij et al. [19] | Publication: 2015  DCE: not reported | NL (HIC) | HepB | Parents of new-borns (<2 wks), not yet eligible for HepB vaccination  Target group: representatives | Study congruence between parents’ SP and RP for vaccination of newborn child against HepB |

| Study | Study characteristics | | | | |
| --- | --- | --- | --- | --- | --- |
|  | **Year** | **Country** | **Vaccine type** | **Study population** | **Objective** |
| Ledent et al. [20] | Publication: 2019  ADCE: 2015-2016 | SP, IT (both HIC) | Tdap | Households and other close contacts of new-borns (<6 mos) or expectant mothers/ partners in last trimester  Target group: vaccinees | Assess relative importance of attributes for pertussis vaccination among households and other close contacts of new-borns and expectant mothers/partners in last trimester, estimate variation in vaccine adoption rate under scenarios (impact cost), assess variation in preferences and uptake across countries and demographics, inform policy and determine which attributes of pertussis cocooning vaccination strategy are important in parental decision making |
| Liao et al. [21] | Publication: 2019  DCE: 2015 | HK (HIC) | Influenza | Adults  Target group: vaccinees | Assess relative effects of altering attributes related to adults’ decision for influenza vaccination choice, assess whether priming modifies relative effects |
| Liao et al. [22] | Publication: 2020 DCE: 2018 | HK (HIC) | Influenza | Healthcare professionals working in public hospitals, eligible for free seasonal influenza vaccination  Target group: health advisors | Examine relative importance of attributes related to vaccine characteristics, social normative influence and convenience in access to influenza vaccine, identify optimal SIV programme for HCPs |
| Lloyd et al. [23] | Publication: 2015  DCE: 2014 | DE (HIC) | Combined hexavalent paediatric vaccines | Paediatricians, nurses (administer ≥15 hexavalent vaccinations monthly, not industry paid/employed)  Target group: health advisors | Reveal HCPs preferences regarding injection devices for hexavalent vaccine programs/explore the importance of different features (or attributes) of fully liquid and non-fully liquid vaccines |
| Marshall et al. [24] | Publication: 2016  DCE: 2013 | AU (HIC) | MenB | Adults, parents, adolescents  Target group: vaccinees and representatives | Explore parent, adolescent and community values for vaccine attributes to assess preferences, assess potential barriers and WTP |
| Ngorsuraches et al. [25] | Publication: 2015  DCE: 2014 | THA (UMIC) | HPV | Parents of at least 1 daughter (9-13 yrs)  Target group: representatives | Assess parents’ preferences for vaccine attributes, calculate WTP |
| Oteng et al. [26] | Publication: 2011  DCE: not reported | CA (HIC) | HPV | Adults (≥19 yrs)  Target group: representatives | Evaluate societal preferences for different vaccination and screening strategies and identify properties of vaccination that are important, determine WTP for attributes |
| Pereira et al. [27] | Publication: 2011  CA: 2010 | USA (HIC) | Influenza | Physicians and clinic managers involved in ordering vaccines  Target group: health advisors | Understand purchase decisions and relative attribute importance in preferences of US clinical office managers and physicians in 43 states |
| Poulos et al. [28] | Publication: 2018  DCE: 2015 | DE (HIC) | Travel | Adult travellers  Target group: vaccinees | Assess importance of selected determinants of travellers’ vaccination choices, examine preferences among different type of travellers, examine if preferences and trade-offs differ between four types of travellers |
| Poulos et al. [29] | Publication: 2015  DCE: 2011 | USA (HIC) | Meningococcal | Paediatricians  Target group: health advisors | Quantify paediatricians’ preferences for vaccine and vaccine features |

| Study | Study characteristics | | | | |
| --- | --- | --- | --- | --- | --- |
|  | **Year** | **Country** | **Vaccine type** | **Study population** | **Objective** |
| Poulos et al. [30] | Publication: 2018  DCE: not reported | PL, HU (both HIC) | Rotavirus | Mothers of children (<3 yrs)  Target group: representatives | Explore importance of mothers’ reasons to vaccinate their child (not related to DCE), explore impact of vaccine attributes on choices, examine whether results vary based on working status of mother |
| Poulos et al. [31] | Publication: 2011  CA: 2009 | VNM (LMIC) | HPV | Mothers with at least 1 daughter (9-17 yrs)  Target group: representatives | Assess WTP for vaccines, estimate trade-offs mothers are willing to make between attributes, measure vaccine coverage for vaccine attributes |
| Sadique et al. [32] | Publication: 2013  DCE: 2007 | UK (HIC) | Hypothetical, based on rotavirus, invasive pneumococcal disease, non-invasive pneumococcal disease | Mothers of children (<5 yrs)  Target group: representatives | Investigate influence of attributes on stated vaccination choices of mothers of young children, investigate trade-offs |
| Sapède et al. [33] | Publication: 2002  CA: not reported | 2 European countries | Hypothetical new vaccines | Parents of children (<21 yrs)  Target group: representatives | 1 objective: explore in two key European countries the perceived value of alternative vaccines and how this translated into WTP among deciders and payers |
| Seanehia et al. [34] | Publication: 2017  CA: 2016 | FR (HIC) | Hypothetical,  similar to MenC and measles | University students (18-24 yrs)  Target group: vaccinees | Quantify preferences among French university students regarding vaccination against severe but rare diseases |
| Shono et al. [35] | Publication: 2014  DCE: 2013 | JPN (HIC) | Influenza | Parents with at least 1 child (<13 yrs)  Target group: representatives | Assess parents’ preferences for seasonal influenza vaccine for their children, evaluate parents’ WTP for vaccine benefits |
| Shono et al. [36] | Publication: 2017  DCE: 2014 | JPN (HIC) | New combination vaccines for children | Mothers with at least one child (2 mos > 2 yrs)  Target group: representatives | Investigate mothers’ preferences for combination vaccines (attributes) for their children |
| Sun et al. [37] | Publication: 2020  CA: 2017 | CHN (UMIC) | Childhood | Parents/caregivers of young infants (<3 mos)  Target group: representatives | Estimate preferences of Chinese parents for vaccine attributes, calculate WTP for attributes |
| Veldwijk et al. [38] | Publication: 2014  DCE: not reported | NL (HIC) | Rotavirus | Parents of newborns (6 wks)  Target group: representatives | Determine parental vaccine preferences, determine potential vaccination coverage for different vaccine scenarios and implementation strategies |
| Verelst et al. [39] | Publication: 2019  DCE: 2017 | ZA (UMIC) | General | Adults (>18 yrs)  Target group: vaccinees and representatives | Explore vaccine decision-making process for general vaccine (for yourself and youngest child) in South-Africa by identifying most influential vaccine attributes, analyse preference heterogeneity, providing policy recommendations |

| Study | Study characteristics | | | | |
| --- | --- | --- | --- | --- | --- |
|  | **Year** | **Country** | **Vaccine type** | **Study population** | **Objective** |
| Verelst et al. [40] | Publication: 2018  DCE: 2017 | BE (HIC) | General | Adults (>18 yrs)  Target group: vaccinees and representatives | Explore determinants of Flemish individuals’ decision-making on vaccination by determining the importance of vaccine attributes, policy and modelling recommendations |
| Wang et al. [41] | Publication: 2017  DCE: 2014-2015 | AU (HIC) | General | Adolescents (15-19 yrs)  Target group: vaccinees | Investigate adolescent preferences to determine most important factors influencing immunization decisions |
| Wong et al. [42] | Publication: 2018  DCE: 2014-2015 | HK (HIC) | HPV | Mothers of daughters (8-17 yrs)  Target group: representatives | Determine consumer preference of vaccine attributes and WTP for HPV vaccine in Hong Kong |

AU = Australia; BE = Belgium; CA = Conjoint analysis; DCE = Discrete Choice Experiment; DE = Germany; GP = General practitioner; HIC = High-Income Country; HPV = Human Papilloma Virus; HU = Hungary; JPN = Japan; LMIC = Low-Middle-Income Country; MenB = Meningococcal B; mos = months; NL = the Netherlands; PL = Poland; SP = Spain; SE = Sweden; UK = United Kingdom; UMIC = Upper-Middle-Income Country; USA = United States of America; wks = weeks; WTP = Willingness-To-Pay; Yrs = years.

**Table 2 Summary of the choice task & experimental design of all included studies**

| Study | Choice task & Experimental design | | |
| --- | --- | --- | --- |
|  | **Methods to create DCE** | **Structure of choice task** | **Experimental design** |
| Adams et al. [1] | 4 methods: literature review, qualitative study, interactive workshop, panel discussions  Create choice sets: D-efficiency, software | Multinomial choice structure  Format: unforced choice with opt-out ('neither'), then forced choice without opt-out (latter only if opt-out was chosen, ‘if vaccination was mandatory') = two-stage choice  Ranking exercise  18 choice tasks | Fractional factorial design  Ngene and exclusion implausible combinations of attribute levels  Main effects |
| Arbiol et al. [2] | 2 methods: literature review, expert interviews and expert group discussions  Create choice sets: fold-over | Multinomial choice structure  Format: unforced choice with opt-out ('no vaccination')  12 choice tasks | Fractional factorial design (orthogonal design)  Catalog approach (orthogonal arrays)  Main and interaction effects |
| Bishai et al. [3] | Methods to identify attributes and create choice sets remain unclear | Multinomial choice structure  Format: forced choice without opt-out, then opt-out provided (actually purchase yes/no) = two-stage choice  18 choice tasks | Fractional factorial design (orthogonal design)  Sawtooth  Not reported (main + interaction effects primary analysis) |
| Brown et al. [4] | 3 methods: literature review, expert consultations, interviews  Create choice sets: unclear | Multinomial choice structure  Format: forced choice without opt-out (text). Example choice question shows third option: opt-out (unforced choice with opt-out described as 'buy neither')  8 choice tasks | Fractional factorial design  Software/approach not reported  Main and interaction effects |
| Brown et al. [5] | 3 methods: literature review, expert consultation, interviews  Create choice sets: unclear | Multinomial choice structure  Format: forced choice without opt-out (text). Example choice question shows third option: opt-out (unforced choice with opt-out described as 'buy neither')  8 choice tasks | Fractional factorial design  Software/approach not reported  Main and interaction effects |
| de Bekker-Grob et al. [6] | 3 methods: literature review, expert interviews, focus groups  Create choice sets: software Street & Burgess | Multinomial choice structure  Format: unforced choice with opt-out (‘no HPV vaccination’)  Ranking exercise  9 choice tasks | Fractional factorial design  Catalog approach (orthogonal arrays)  Main and interaction effects |
| de Bekker-Grob et al. [7] | 3 methods: literature review, expert interviews, focus groups  Create choice sets: D-efficiency | Multinomial choice structure  Format: unforced choice with opt-out (‘no vaccination’)  16 choice tasks (+ 1 warm-up question) | Fractional factorial design (heterogeneous DCE design)  Bayesian efficient design algorithms  Main and interaction effects |
| Determann et al. [8] | 3 methods: literature review, expert interviews, focus groups  Create choice sets: D-efficiency, software (Ngene) | Multinomial choice structure  Format: unforced choice with opt-out (‘no vaccination’)  Ranking exercise  16 choice tasks | Fractional factorial design  Ngene  Main and interaction effects |
| Determann et al. [9] | 3 methods: literature review, expert interviews, focus groups  Create choice sets: D-efficiency, software (Ngene) | Multinomial choice structure  Format: unforced choice with opt-out (‘no vaccination’)  16 choice tasks | Fractional factorial  Ngene  Main and interaction effects |

| Study | Choice task & Experimental design | | |
| --- | --- | --- | --- |
|  | **Methods to create DCE** | **Structure of choice task** | **Experimental design** |
| Eilers et al. [10] | 2 methods: literature review, focus group study  Create choice sets: D-efficiency, software (Ngene) | Multinomial choice structure  Format: unforced choice with opt-out (‘no vaccination’)  6 choice tasks | Fractional factorial design  Ngene  Main and interaction effects |
| Flood et al. [11] | 3 methods: literature review, qualitative research, expert interviews  Create choice sets: unclear | Multinomial choice structure  Format: forced choice without opt-out  Ranking exercise  Number of choice tasks remain unclear | Experimental design unclear  Software/approach not reported  Not reported (unclear from analysis) |
| Flood et al. [12] | 3 methods: literature review, parent interviews, expert consultation  Create choice sets: unclear | Multinomial choice structure  Format: forced choice without opt-out  Rating exercise (5-point scale)  Number of choice tasks remain unclear | Experimental design unclear  Software/approach not reported  Not reported (unclear from analysis) |
| Gidengil et al. [13] | 2 methods: literature review, interviews with paediatricians, community members and policy makers  Create choice sets: unclear | Multinomial choice structure  Format: forced choice without opt-out, then opt-out (ask if respondents would vaccinate their child in real life) = two-stage choice  17 choice tasks | Fractional factorial design  Software/approach not reported  Not reported (main effects in primary analysis, interaction terms included in appendix) |
| Guo et al. [14] | 2 methods: literature review, expert consultation  Create choice sets: unclear | Multinomial choice structure  Format: unforced choice with opt-out (‘no vaccination’), then forced choice without opt-out (‘if you were actually offered the 2 vaccines above, which would you prefer to choose?’) = two-stage choice  6 choice tasks | Fractional factorial design (orthogonal design)  Software/approach not reported  Main and interaction effects |
| Hall et al. [15] | 2 methods: literature review, expert consultation  Create choice sets: unclear | Binary choice structure (1 scenario provided)  Format: yes/no  16 choice tasks | Fractional factorial design (orthogonal)  Software/approach not reported  Main and interaction effects |
| Hofman et al. [16] | 3 methods: literature review, focus groups, expert interviews  Create choice sets: D-efficiency, software (SAS) | Multinomial choice structure  Format: unforced choice with opt-out (‘no vaccination)  Ranking exercise  9 choice tasks | Fractional factorial design  SAS  Main and interaction effects |
| Hofman et al. [17] | 2 methods: previous DCE, literature (CDC report, quantitative study)  Create choice sets: D-efficiency, software (Ngene) | Multinomial choice structure  Format: unforced choice with opt-out (’no HPV vaccination’)  Ranking exercise  16 choice tasks | Fractional factorial design  Ngene  Main and interaction effects |
| Huang et al. [18] | 3 methods: previous DCE, qualitative study  Create choice sets: software(SAS) | Multinomial choice structure  Format: forced choice without opt-out  4 choice tasks (+ 1 example) | Fractional factorial design  SAS  Not reported (main effects primary analysis) |
| Lambooij et al. [19] | 3 methods: qualitative research, literature review, expert interviews  Create choice sets: fold-over | Multinomial choice structure  Format: forced choice without opt-out, then opt-out (indicate on 10-point scale how certain choice to vaccinate child is)  4 choice tasks | Full factorial design  Software/approach not reported  Not reported (main effects primary analysis) |
| Study | Choice task & Experimental design | | |
|  | **Methods to create DCE** | **Structure of choice task** | **Experimental design** |
| Ledent et al. [20] | 1 method: focus group  Create choice sets: software | Multinomial choice structure  Format: forced choice without opt-out, later opt-out provided (‘how likely would you be to get this vaccine?’ Indicated on 5-point scale) = two-stage choice  Number of choice tasks remain unclear | Experimental design unclear (only ‘questionnaire showed near-complete orthogonality, minimal overlap with level balance’)  Sawtooth  Not reported (main effects primary analysis) |
| Liao et al. [21] | 2 methods: theories, literature review  Create choice sets: random | Multinomial choice structure  Format: unforced choice with opt-out (‘neither A nor B’)  8 choice tasks (+ 1 for rationality test) | Fractional factorial design  SPSS (orthogonal arrays)  Main and interaction effects |
| Liao et al. [22] | 2 methods: longitudinal survey, realistic and meaningful principle Create choice sets: unclear | Multinomial choice structure  Format: unforced choice with opt-out (neither A nor B)  8 choice tasks (+ 1 for rationality test) | Fractional factorial design  Catalog approach (orthogonal arrays)  Not reported (main effects primary analysis) |
| Lloyd et al. [23] | 2 methods: literature review, qualitative research (interviews HCPs)  Create choice sets: fold-over | Multinomial choice structure  Format: not reported  Number of choice tasks remain unclear | Fractional factorial design  Software/approach not reported  Main and interaction effects |
| Marshall et al. [24] | 2 methods: literature review, expert consultation  Create choice sets: software (Ngene) | Multinomial choice structure  Format: forced choice without opt-out, then opt-out (indicate if you would choose to be vaccinated in preferred option from forced choice) = two-stage choice  12 choice tasks (2 dominant options for rationality test) | Fractional factorial design (sequential orthogonal factorial design)  Ngene  Not reported (main effects primary analysis) |
| Ngorsuraches et al. [25] | 1 method: literature review  Create choice sets: random, software (Ngene) | Multinomial choice structure  Format: unforced choice task with opt-out (‘no vaccination’)  6 choice tasks (+ 1 for dominance test) | Fractional factorial design (orthogonal design)  Ngene  Not reported (main effects primary analysis) |
| Oteng et al. [26] | 4 methods: vaccination and screening policy, literature review, qualitative survey, expert consultation  Create choice sets: software (sawtooth) | Multinomial choice structure  Format: unforced choice with opt-out (‘neither’)  10 choice tasks (+ 2 for consistency test) | Fractional factorial design  Sawtooth  Not reported (main effects primary analysis) |
| Pereira et al. [27] | 2 methods: literature review, interviews with industry and HC professionals  Create choice sets: unclear | Multinomial choice structure  Format: forced choice without opt-out  9 choice tasks (+ 1 warm up question) | Fractional factorial design (orthogonal design)  Software/approach not reported  Not reported (main effects primary analysis) |
| Poulos et al. [28] | 1 method: based disease risk and vaccine characteristics (literature used?)  Create choice sets: D-optimal iterative computer algorithm (d-efficiency, software) | Multinomial choice structure  Format: forced choice without opt-out, then unforced choice with opt-out (indicate if selected vaccine a/b is preferred or preference for ‘no vaccine’) = two-stage choice  9 choice tasks | Fractional factorial design.  D-optimal iterative computer algorithm (software)  Main and interaction effects |

| Study | Choice task & Experimental design | | |
| --- | --- | --- | --- |
|  | **Methods to create DCE** | **Structure of choice task** | **Experimental design** |
| Poulos et al. [29] | 1 method: literature review  Create choice sets: D-efficiency, software (SAS) | Multinomial choice structure  Format: unforced choice with opt-out (‘neither’)  9 choice tasks | Fractional factorial design.  SAS  Main and interaction effects |
| Poulos et al. [30] | 2 methods: characteristics of vaccines, qualitative interviews/discussions  Create choice sets: unclear | Multinomial choice structure  Format: unforced choice with opt-out (‘neither vaccine’)  Rating exercise  9 choice tasks | Experimental design unclear  Software/approach not reported  Main and interaction effects |
| Poulos et al. [31] | 4 methods: qualitative research, interviews, collaborators familiar with local conditions (expert consultation), previous DCEs  Create choice sets: unclear | Multinomial choice structure  Format: unforced choice with opt-out (‘neither’)  6 choice tasks | Experimental design unclear  Software/approach remain unclear (variation of commonly used algorithm)  Not reported (main effects primary analysis, interaction terms included in appendix) |
| Sadique et al. [32] | 1 method: literature review  Create choice sets: fold-over design | Binary choice structure  Format: yes/no  Rating exercise  Best-worst scaling exercise  9 choice tasks (+ 3 regret + 1 for rationality test) | Fractional factorial design  Software/approach not reported  Not reported (main effects primary analysis) |
| Sapède et al. [33] | 1 method: focus group  Create choice sets: unclear | Multinomial choice structure  Format: forced choice without opt-out, later opt-out (actually buy preferred vaccine if it available?) = two stage choice  Rating exercise  36 choice tasks | Experimental design unclear Software/approach: not reported  Not reported (unclear from analysis) |
| Seanehia et al. [34] | 1 method: literature review  Present scenarios: random | Binary choice structure  Format: yes/no, if vaccine accepted then indicate maximum minor side effect to maintain acceptance (choice between 3) = two-stage choice  24 choice tasks | Fractional factorial design  Approach: manual based on exclusion impossible scenarios, random exclusion and exclusion scenarios with each level of given attribute (no software)  Not reported (main effects primary analysis) |
| Shono et al. [35] | 1 method: literature review  Create choice sets: random | Multinomial choice structure  Format: unforced choice with opt-out (‘neither vaccination’)  5 choice tasks | Fractional factorial design  Catalog approach (library orthogonal arrays)  Main and interaction effects |
| Shono et al. [36] | 1 method: literature review  Create choice sets: random | Multinomial choice structure  Format: unforced choice with opt-out (‘no vaccination’)  5 choice tasks | Fractional factorial design  Catalog approach (library orthogonal arrays)  Main and interaction effects |
| Sun et al. [37] | 1 method: qualitative research  Create choice sets: unclear | Multinomial choice structure  Format: forced choice without opt-out  Number of choice tasks remain unclear | Fractional factorial design  Software/approach not reported  Main and interaction effects |
| Veldwijk et al. [38] | 3 methods: literature review, group and expert interviews  Create choice sets: D-efficiency, software (Ngene) | Multinomial choice structure  Format: forced choice without opt-out, then opt-out (indicate if same choice is made in real life) = two-stage choice  9 choice tasks | Fractional factorial design  Ngene  Main and interaction effects |

| Study | Choice task & Experimental design | | |
| --- | --- | --- | --- |
|  | **Methods to create DCE** | **Structure of choice task** | **Experimental design** |
| Verelst et al. [39] | 2 methods: literature review, previous DCE  Create choice sets: D-efficiency | Multinomial choice structure  Format: forced choice without opt-out  10 choice tasks | Fractional factorial design (Bayesian optimal design)  Multivariate normal prior distribution reflecting prior beliefs unknown parameter values (exp. interviews, lit. review)  Main and interaction effects |
| Verelst et al. [40] | 2 methods: literature review, focus groups  Create choice sets: D-efficiency (D-optimal) | Multinomial choice structure  Format: forced choice without opt-out  10 choice tasks (+1 illustrative example) | Fractional factorial design (Bayesian optimal design)  Multivariate normal prior distribution reflecting prior beliefs unknown parameter values (exp. interviews, lit. review)  Main and interaction effects |
| Wang et al. [41] | 2 methods: literature review, expert consultation  Create choice sets: D-efficiency, software (Ngene) | Multinomial choice structure  Format: forced choice without opt-out  12 choice tasks (+ 1 repeated for consistency test) | Fractional factorial design  Ngene  Not reported (main effects in primary analysis) |
| Wong et al. [42] | 2 methods: literature review, expert interviews  Create choice sets: software (SPSS) | Multinomial choice structure  Format: unforced choice with opt-out (‘no vaccination’)  8 choice tasks (+ 1 for rationality test) | Fractional factorial design (orthogonal design)  SPSS  Main effects |

DCE = Discrete Choice Experiment; exp. interviews = expert interviews; HCPs = healthcare providers; lit. review = literature review.

**Table 3 Summary of the way in which all included studies were conducted**

| Study | Conduct | | | |
| --- | --- | --- | --- | --- |
|  | **Mode of administration** | **Piloting/pre-testing** | **Sample size** | **(Financial) compensation** |
| Adams et al. [1] | Self-administered  Completed online | Yes, paper pilot (n=5), electronic pilot: first and second soft launch (resp. n=40 and n=77), both among parents of pre-school children | n=521 (at risk n=259 at risk, not at risk n=262)  Justification of sample size included (rule of thumb) | Yes, money  (£1-2) |
| Arbiol et al. [2] | Interview-administered | Yes, no further details reported | n=342  No justification nor sample size calculations | Unclear |
| Bishai et al. [3] | Not reported | Not reported | n=229  No justification nor sample size calculations | Unclear |
| Brown et al. [4] | Self-administered  Completed online | Yes, interviews with mothers with daughters aged 13-17 yrs (n=30) | n=307  Justification of sample size included (rule of thumb) | Unclear |
| Brown et al. [5] | Self-administered  Completed online | Yes, interviews with daughters aged 13-17 yrs (n=30) | n=307  Justification of sample size included (rule of thumb) | Unclear |
| de Bekker-Grob et al. [6] | Self-administered  Probably paper based  Classroom/auditorium with assistant | Yes (n=16) | n=312  Justification of sample size included (rule of thumb) | Unclear |
| de Bekker-Grob et al. [7] | Self-administered  Completed online | Yes, qualitative pre-pilot study using think-aloud strategy (n=20) and pilot (n=300) | n=1261  Justification of sample size included (sample size calculations) | Yes, money (€2.20) |
| Determann et al. [8] | Self-administered  Completed online | Yes, paper-based pilot (n=29) and think-a-loud interviews (n=5) | n=536  Justification of sample size included (rule of thumb) | Yes, money for focus group €40), for DCE (€2.20) |
| Determann et al. [9] | Self-administered  Completed online | Yes, pen-and-paper pilot conducted in NL (n=29) and think-a-loud interviews (n=5) | n=2068 (n=536 NL, n=512 SP, n=510 PL, n=510 SE)  No justification nor sample size calculations | Yes, amount depends on country (e.g. €2.20 in NL) |
| Eilers et al. [10] | Self-administered  Paper-based | Yes, think out loud testing among persons aged 52>82 yrs (n=8) | n=610  No justification nor sample size calculations | Yes, voucher (€10) |
| Flood et al. [11] | Self-administered  Completed online | Yes, pilot among small sample (no further details) | n=464  No justification nor sample size calculations | Unclear |
| Flood et al. [12] | Self-administered  Completed online | Not reported | n=451  No justification nor sample size calculations | Unclear |
| Gidengil et al. [13] | Self-administered  Completed online | Yes (n=57) | n=558  No justification nor sample size calculations | Unclear |
| Guo et al. [14] | Interview administered | Not reported | n=266  Justification of sample size included (rule of thumb) | Unclear |
| Hall et al. [15] | Interview administered  By telephone | Yes, feasibility study (no further details) | n=50  No justification nor sample size calculations | Unclear |
| Hofman et al. [16] | Self-administered  Paper-based | Yes (n=16) | n=294  Justification of sample size included (rule of thumb) | Unclear |

| Study | Conduct | | | |
| --- | --- | --- | --- | --- |
|  | **Mode of administration** | **Piloting/pre-testing** | **Sample size** | **(Financial) compensation** |
| Hofman et al. [17] | Self-administered  Paper-based  In classroom, auditorium | No pilot, justification lack of pilot included | n=500  Justification of sample included (rule of thumb) | Unclear |
| Huang et al. [18] | Self-administered  Paper-based | Not reported | n=590  Justification of sample size included (rule of thumb) | Unclear |
| Lambooij et al. [19] | Self-administered  Paper-based | Not reported | n=896  No justification nor sample size calculations | Unclear |
| Ledent et al. [20] | Not reported | Yes, Spain (n=50) and Italy (n=50) in 2014-2015 | n=615  Justification of sample size included (rule of thumb) | No |
| Liao et al. [21] | Self-administered  Completed on tablet | Yes, no further details reported | n=800  No justification nor sample size calculations | Unclear |
| Liao et al. [22] | Self-administered or interview administered (preference participant) | Yes, no further details reported | n=258  No justification nor sample size calculations | Unclear |
| Lloyd et al. [23] | Self-administered  Completed online | Yes, among HCPs (n=2 paediatricians, n=2 nurses) | n=500 (n=150 nurses, n=150 physicians)  No justification nor sample size calculations | Yes, small recommended fee |
| Marshall et al. [24] | Self-administered  Completed online | Yes, conducted in 2013 among 57 adolescents and 120 adults (n=177) | n=2505 (n=502 adolescents, n= 2003 adults)  No justification nor sample size calculations | Unclear |
| Ngorsuraches et al. [25] | Self-administered  No further details | Yes, think aloud testing (n=5) and pilot (n=30) | n=314 (fathers n=150, mothers n=164)  Justification of sample size included (rule of thumb) | Unclear |
| Oteng et al. [26] | Self-administered  Completed online | Yes (n=300) | n=1157  No justification nor sample size calculations | Unclear |
| Pereira et al. [27] | Self-administered  Completed online | Not reported | n=235 (sees adults n=88, only children n=147)  Justification of sample size is included (power calculations) | Yes, Amazon giftcard ($25) |
| Poulos et al. [28] | Self-administered  Completed online | Yes, pre-test among individuals with previous/planned international travel (n=15) | n=603 (n=148 business, n=153 visiting friends/family, n=152 leisure, n=150 backpack)  No justification nor sample size calculations | Yes, money (€25) |
| Poulos et al. [29] | Self-administered  Completed online | Yes, semi-structured interviews with paediatricians (n=10) | n=214  No justification nor sample size calculations | Yes, money ($55) |
| Poulos et al. [30] | Self-administered  Completed online | Yes, pre-test among mothers of children < 3 yrs (n=15) | n=700 (n=350 Poland, n=350 Hungary)  No justification nor sample size calculations | Unclear |
| Poulos et al. [31] | Self-administered  Paper-based | Yes, pre-test among mothers (n=50) and pilot (no further details) | n=258  No justification nor sample size calculations | Unclear |
| Sadique et al. [32] | Interview administered | Yes, among mothers (n=15) | n=369  No justification nor sample size calculations | No |
| Sapède et al. [33] | Interview administered | Yes, no further details reported | n=229 (n=115 country A, n=114 country B), Justification of sample size included (rule of thumb) | Unclear |

| Study | Conduct | | | |
| --- | --- | --- | --- | --- |
|  | **Mode of administration** | **Piloting/pre-testing** | **Sample size** | **(Financial) compensation** |
| Seanehia et al. [34] | Self-administered  Completed online  Link in e-mail | Yes, among students in two public health schools | n=775  Justification of sample size included (rule of thumb) | Yes, lottery of cash vouchers (€15) |
| Shono et al. [35] | Self-administered  Completed online | Yes, no further details reported | n=555  No justification nor sample size calculations | Yes, reward transferable in giftcard (￥33) |
| Shono et al. [36] | Self-administered  Completed online | Yes, pre-test among individuals with at least one child (n=12) | n=1243  Justification of sample size included (rule of thumb) | Unclear |
| Sun et al. [37] | Self-administered  Paper-based  Completed in private room | Not reported | n=552  Justification of sample size included (rule of thumb) | Yes, small gift (e.g. umbrella or blanket) |
| Veldwijk et al. [38] | Self-administered  Paper-based | Yes, among parents of new-borns (n=48), four tests were think aloud tests | n=466  No justification nor sample size calculations | Unclear |
| Verelst et al. [39] | Self-administered  Completed online | Only soft launch in small sample of panel | n=1200 (adult group n=600, child group n=600)  Justification of sample size included (rule of thumb) | Yes, credit rewards transferable into giftcards, coupons, airmiles |
| Verelst et al. [40] | Self-administered  Completed online | Yes, also soft launch in population (no further details on pilot) | n=1919 (n=1091, adult group, child n=828 child group)  Justification of sample size included (rule of thumb) | Yes, credit rewards transferable into giftcard, coupons, airmiles |
| Wang et al. [41] | Self-administered  Completed online | Yes, pilot (n=130) and pre-pilot (n=3) | n=695  Justification of sample size included (rule of thumb) | Yes, money (AU$3.25) |
| Wong et al. [42] | Self-administered  Completed online  Laptop/tablet, assistant available for help | Yes, performed in 2017 (n=8 paediatricians, n=8 mothers) | n=482  Justification of sample size included (rule of thumb) | Unclear |

**Table 4 Summary of the way in which data of included studies were analysed**

| Study | Analysis | | | |
| --- | --- | --- | --- | --- |
|  | **Estimation procedure** | **Subgroup analysis** | **Outcome measures** | **Analysis software** |
| Adams et al. [1] | Mixed logit | Yes, split sample (separate models) based on risk: 'at high risk' and 'not at high risk for incomplete vaccination'  Determined by 5 sociodemographic factors: living area, mental state, marital status, age, number of children | Marginal WTA  Vaccine uptake/probability analysis | Not reported |
| Arbiol et al. [2] | Random parameters logit | Yes, split sample (separate models) based on health status: leptospirosis vs. non-leptospirosis  Additional subgroups based on 8 sociodemographic factors (age, education, family size, income, gender, living near to market, living near to river, living near to sewer) and 1 factor related to awareness of leptospirosis | (Marginal) WTP | Nlogit |
| Bishai et al. [3] | Conditional logit,  Generalised linear-random effect logit | Yes, split sample (separate models) based on country (sociodemographic factor) and video exposure: Germany-France, video-no video  Subgroups related to objective  Comparison of respondents who passed/failed dominance test  Additional subgroups based on 1 sociodemographic factor (income), 2 related to knowledge/perception/awareness (perceived risk meningitis without vaccine, knowledge serogroup) | Probability of purchase (probability analysis) | Stata |
| Brown et al. [4] | Mixed logit | Yes, based on sociodemographic factors (age, race/ethnicity, household income, education), factors targeting perception (concern HPV/cervical cancer/genital warts risks), beliefs (risks and safety), previous vaccination status, previous diagnosis HPV/cervical cancer/genital warts  Related to objective  Comparison of respondents who passed/failed consistency test | WTP  Vaccine uptake under different policy scenarios | Stata |
| Brown et al. [5] | Latent class logit,  Mixed logit | Yes, split sample (separate models) based on 2 classes which differ price sensitivity and preference for higher/improved attribute levels  Subgroups related to econometric model and objective  Additional subgroups based on sociodemographic factors (age, race, house income, personal income), interest in vaccine to a parent, willingness/intention to spend on vaccine, factors related to attitude/health experiences/beliefs (previous pap test, concern of risk HPV/cervical cancer/genital warts, beliefs about safety)  Subgroups related to objective  Comparison of respondents failed/passed consistency test | WTP  Vaccine uptake under different policy scenarios | Stata |
| de Bekker-Grob et al. [6] | Mixed logit | Yes, comparison of respondents who failed/passed dominance test | Vaccination uptake analysis  Substitution/trade-off rate | Not reported |
| de Bekker-Grob et al. [7] | (Heteroscedastic) multinomial logit,  Random intercept | Yes, based on 19 characteristics (interaction terms): 8 sociodemographic factors, 8 factors related to vaccination attitude, intention, belief, vaccination status, impact condition on family, 3 related to decision-making skills (style, health literacy, numeracy)  Subgroups related to objective | Vaccine uptake/probability analysis (choice probabilities) | Pythonbiogeme software |

| Study | Analysis | | | |
| --- | --- | --- | --- | --- |
|  | **Estimation procedure** | **Subgroup analysis** | **Outcome measures** | **Analysis software** |
| Determann et al. [8] | Latent class (panel version) | Yes, split sample (separate models) based on 2 classes depending on 1 sociodemographic factor (sex) and 1 related to attitude (towards vaccination)  Classes related to econometric model  2 additional scenarios identified: mild and severe pandemic defined by severity and susceptibility of disease (interaction terms)  Scenarios based on objective | Relative importance scores  WTP  Uptake/probability analysis | Nlogit  SPSS |
| Determann et al. [9] | Panel latent class regression | Yes, split sample (separate models) based on 3 classes partly depending on country  Classes related to econometric model and objective. | Relative importance scores  Vaccine uptake analysis under pandemic scenarios | Nlogit  SPSS |
| Eilers et al. [10] | Mixed multinomial logit | Yes, split sample (separate models) based on sociodemographic factor age (50-65 yrs, >65 yrs)  Additional subgroups based on 2 sociodemographic factors (gender, education) and factors related to vaccination status and health status (separate models for latter two factors) | Vaccine acceptance analysis | Nlogit  SAS |
| Flood et al. [11] | Multinomial logit,  Hierarchial Bayes | Yes, based on 2 sociodemographic factors (age, gender) and vaccine behaviour parents | Relative attribute importance (utility estimates) in scenarios  Market simulation | Sawtooth  SAS |
| Flood et al. [12] | Multinomial logit, Hierarchical Bayes | Yes, based on 5 sociodemographic factors (age child, gender, household income, education, race/ethnicity), vaccination intention, preference for certain type of vaccine administration  Subgroups related to objective | Relative attribute importance (utility estimates and ratings) under scenarios | Sawtooth  SAS |
| Gidengil et al. [13] | Multivariate logistic regression with generalized estimating equations | Yes, split sample (separate models) based on parental status (yes/no).  Additional subgroups based on 6 sociodemographic factors (age, gender, race, education, income, insurance status), perceived safety, preference for splitting up vaccines between visit  Latter subgroup based on objective  Comparison of respondents who passed/failed dominance test  Comparison of respondents with and without preference for opt-out | WTP (TTO and WTP in direction valuation) | SAS |
| Guo et al. [14] | Mixed logit | Yes, based on 1 sociodemographic factor (SES) and 3 disease related factors (type of disease, susceptibility and severity of disease), interaction terms included for subgroups | WTP  Vaccine uptake/probability analysis | Stata |
| Hall et al. [15] | Random effects logit | Yes, based on 1 sociodemographic factor (country of origin/birth) | Vaccine uptake under different program scenarios | SAS |
| Hofman et al. [16] | Panel mixed logit regression | Yes, comparison of respondents who failed/passed dominance test | Vaccine uptake under specific scenarios  Trade-offs/substitution rate | Not reported |

| Study | Analysis | | | |
| --- | --- | --- | --- | --- |
|  | **Estimation procedure** | **Subgroup analysis** | **Outcome measures** | **Analysis software** |
| Hofman et al. [17] | Panel latent class | Yes, split sample (separate models) based on 3 classes depending on one sociodemographic factor (education) and vaccination status  Classes related to econometric model (methodology)  Comparison of respondents who passed/failed rationality test | Substitution/trade-off vaccine attributes | Nlogit |
| Huang et al. [18] | Conditional logistic regression | Yes, based on 3 sociodemographic factors (parental role, education, income) | Odds ratio  WTP | SAS |
| Lambooij et al. [19] | Mixed logit | No | Individual utility scores  Comparison predictions (estimated individual utility) and actual behaviour (database info) expressed in positive or negative predictive value | Nlogit |
| Ledent et al. [20] | Multinomial logit,  Hierarchical Bayes | Yes, split sample (separate models) based on sociodemographic factor country (Spain, Italy)  Subgroups related to objective  Additional subgroups based on sociodemographic factors gender, age, education  Subgroups related to objective | Individual utility scores (part-worth utilities)  Vaccine uptake/probability analysis | Sawtooth  SAS |
| Liao et al. [21] | Mixed logit | Yes, split sample (separate models) based on (types of) video exposure: control/no video, influenza risk, vaccination risk and air-pollution risk (priming condition)  Subgroups related to objective  Additional subgroups based on pandemic severity (2 types: A/H1N1-like pandemic and A/H5N1-like pandemic)  Comparison of respondents who passed/failed rationality test | Relative attribute importance under conditions/scenarios | R version 3.4.0 (Foundation for statistical computing platform, 2017) |
| Liao et al. [22] | Mixed logit regression | Yes, based on ones who passed/failed the rationality test | Relative attribute importance (preference weights)  WTT  Vaccine uptake/probability analysis | Stata |
| Lloyd et al. [23] | Conditional logit | Yes, split sample (separate models) based on profession (physicians, nurses)  Subgroups related to target group  Additional subgroups based on 2 sociodemographic factors profession (nurse, physician) and current role (administer only vs prepare and administer/prepare only), 1 factor related to previous experience 'HCP vaccine preference' (non-fully-liquid vaccine vs fully liquid vaccine) | Odds ratio | SAS |
| Marshall et al. [24] | Mixed logit regression | Yes, split sample (separate models) based on age and parental status (adolescents vs. adults, adults with children under 18 yrs vs. adults without children under 18 yrs)  Subgroups related to objective | WTP | Stata |
| Ngorsuraches et al. [25] | Multinomial logit | Yes, split sample (separate models) based on sociodemographic factor gender (mother-father) | WTP | Nlogit |
| Study | Analysis | | | |
|  | **Estimation procedure** | **Subgroup analysis** | **Outcome measures** | **Analysis software** |
| Oteng et al. [26] | Mixed effect logit | Yes, based on 7 sociodemographic factors (sex, age, education, income, parental status, household, sexual activity child) and 1 factor related to experience (HPV experience)  Comparison of respondents passed/failed consistency test (only in terms of sociodemographic factors) | WTP  WTT | SAS  Matlab code |
| Pereira et al. [27] | Random effects logistic regression | Yes, split sample (separate models) based on work setting (primarily sees adults-primarily sees children)  Additional subgroups based on 2 sociodemographic factors (degree/education, geographic location) | Change in log odds for attributes  WTP | Stata |
| Poulos et al. [28] | Mixed logit,  Multivariate logit regression | Yes, split sample (separate models) based on type of traveller (business, visiting friends/family, leisure, backpack)  Subgroups related to objective  Scope test: 2 different cost ranges provided to groups (split sample)  Additional subgroups based on 7 sociodemographic factors (gender, age, partnership, children, living situation, income, education), 2 for vaccination status (has/will receive a travel vaccine, chose not to get recommended vaccine), factor about concern (infecting others), 1 on importance of duration, 3 on previous experience (bought vaccine, sick on travel, preventable illness) | Odds ratio, change in log odds  2 types of MME  Trade-off | Nlogit |
| Poulos et al. [29] | Random parameters logit regression,  Conditional logit | Yes, split sample due to scope test: different cost ranges used (narrow: $0-75 vs. wide: $0-150) and 2 different information/risk communication formats used (constant vs variable-base population information format) | Change in log-odds  Substitution rate (MAE) | Nlogit, 500 draws from Halton sequence |
| Poulos et al. [30] | Random parameter logit | Yes, split sample (separate models) based on sociodemographic factor country (Poland-Hungary).  Additional subgroups based on 1 sociodemographic factor (working status)  Subgroups related to objective  Scope test: 2 different cost ranges used (narrow-wider with each 4 levels) | Change in log odds (preferences weights and preference modelling)  Monetary equivalents/WTP | Not reported |
| Poulos et al. [31] | Conditional logit,  Robust variance estimator | Yes, based on sociodemographic factor (SES) | Probability analysis (predicted choice probabilities)  WTP | Stata |
| Sadique et al. [32] | Logistic regression (panel structure),  Random effects | Yes, based on 4 socio-demographic factors (SES, income, education, ethnicity) and 2 factors related to perceptions and beliefs (perceptions of severity of rotavirus infection, immunisation weakens immunity) | WTP/WTA  Probability/uptake analysis | Stata |
| Sapède et al. [33] | Choice-based conjoint-hierarchical Bayes | Yes, split sample (separate models) based on sociodemographic factor country (A, B) and based on educational material seen (video/no video) | Individual and average utility scores (incl. relative attribute importance) Vaccine uptake  Market simulation with revenue calculation | Sawtooth  SPSS |

| Study | Analysis | | | |
| --- | --- | --- | --- | --- |
|  | **Estimation procedure** | **Subgroup analysis** | **Outcome measures** | **Analysis software** |
| Seanehia et al. [34] | Panel logit,  Ordered panel logit,  Random effect estimator logit | Yes, split sample (separate models) for 3 sociodemographic factors (gender, academic discipline, professional category mother) and 2 targeting health behaviour (use of alternative medicine and source vaccination info)  Also split sample (separate models) based on attitude/vaccine perceptions and trust  Comparison of ordinary and binary outcomes | Odds ratio | Stata |
| Shono et al. [35] | Conditional logit | Yes, separate model for interaction terms based on 5 sociodemographic factors (children, household income, age, sex, education), vaccination status (previous uptake vaccine child), 2 targeting previous experience (influenza experience child, experience AE) | WTP | Not reported |
| Shono et al. [36] | Mixed logit | Yes, separate model for interaction terms based on 4 sociodemographic factors (employment, no of children, household income, age child) | WTP | Not reported |
| Sun et al. [37] | Logistic regression | Yes, based on 3 sociodemographic factors (income, residency, education), in sensitivity analysis | Odds ratio | SAS |
| Veldwijk et al. [38] | Mixed-logit incl. random effects | Yes, split sample (separate models) based on vaccination intention, 2 targeting vaccine perception (perceived severity and susceptibility) | Vaccination uptake analysis  Willingness to trade attributes | Not reported |
| Verelst et al. [39] | Panel mixed logit,  Hierarchical Bayes | Yes, split sample (separate models) based on 2 panels (decision adult or child)  Subgroups related to objective  Interaction terms included for subgroups based on sociodemographic characteristics (province, internet access, occupational status, household, religion), vaccine attitudes, risk perception (relier, thinker).  Ethnicity adjusted analysis performed | Relative attribute importance (expressed in logworth statistic and marginal utilities) | JMP Pro 13 (choice platform) |
| Verelst et al. [40] | Panel mixed logit, Hierarchical Bayes | Yes, split sample (separate models) based on 2 panels (decision adult or child)  Panels related to objective  Additional subgroups based on sociodemographic factors (age), vaccine attitudes, risk perception (source of information, acceptor) | Relative attribute importance (logworth statistic and marginal utilities) | JMP 13 pro |
| Wang et al. [41] | Mixed logit | Yes, based on sociodemographic factor (SES), risk taking attitude and vaccination intention  Comparison of respondents who passed/failed the consistency test | Relative attribute importance  WTP | SEIFA  Stata |
| Wong et al. [42] | Multinomial logistic regression | Yes, based on 2 sociodemographic factors (education, monthly household income), separate models for WTP | Marginal and overall WTP | SAS |

AE = Adverse Event; MME = Mean Monetary Equivalents; WTA = Willingness-To-Accept; WTP = Willingness-To-Pay; WTT = Willingness-To-Trade; yrs = years.

**Table 5 Summary of the other (remaining) characteristics of all included studies**

| Study | Other | | |
| --- | --- | --- | --- |
|  | **Journal** | **Source of funding** | **Additional eligible studies** |
| Adams et al. [1] | Health Technology Assessment | Funded by National Institute for Health Research | No |
| Arbiol et al. [2] | Human Vaccines & Immunotherapeutics | Funded by the Leptospirosis Prevention and Control Program in the Philippines | No |
| Bishai et al. [3] | Pharmacoeconomics | Research grant of Sanofi Pasteur | No |
| Brown et al. [4] | Vaccine | Funded by the Centers for Disease Control and Prevention, contract # 200-2002-00776TO43 with RTI International | No |
| Brown et al. [5] | Advances in Health Economics and Health Services Research | Funded by the Centers for Disease Control and Prevention, contract # 200-2002-00776TO43 and 0211878 with RTI International | No |
| de Bekker-Grob et al. [6] | Vaccine | Grant from the Dutch Cancer Society (no. EMCR 2008-3992) | No |
| de Bekker-Grob et al. [7] | Vaccine | Grant from the Netherlands Organisation for Scientific Research (NWO-Talent-Scheme-Veni-Grant No. 451-15- 039) | No |
| Determann et al. [8] | PLoS One | Grant from the European Union Seventh Framework Programme (FP7/2007-2013), no. 278763 | No |
| Determann et al. [9] | Eurosurveillance | Grant from the European Union Seventh Framework Programme (FP7/2007-2013), no. 278763 | No |
| Eilers et al. [10] | Vaccine | Funded by the Dutch Ministry of Health, Welfare and Sport | No |
| Flood et al. [11] | Vaccine | Funded by MedImmune, LLC, manufacturer of an influenza vaccine | No |
| Flood et al. [12] | Clinical Pediatrics | Funded by MedImmune, LLC, manufacturer of an influenza vaccine | No |
| Gidengil et al. [13] | Vaccine | Funded by the cooperative agreement U01 IP000143-01 from Centers for Disease Control and Prevention, National Center for Immunization and Respiratory Diseases | No |
| Guo et al. [14] | Vaccine | Grant from China Postdoctoral Science Foundation (Grant number 2015M570908) | No |
| Hall et al. [15] | Health Economics | Research grant from Medical Foundation of the University of Sydney | No |
| Hofman et al. [16] | BMC Public Health | Grant from the Dutch Cancer Society (no. EMCR 2008–3992) | No |
| Hofman et al. [17] | PLoS One | Funded by the Dutch Cancer Society (no. EMCR 2009-4561) | No |
| Huang et al. [18] | Human Vaccines & Immunotherapeutics | Funded by the Fourth Round of Three-Year Public Health Action Plan of Shanghai, China (No. 15GWZK0101) | No |
| Lambooij et al. [19] | BMC Medical Research Methodology | Not reported | No |
| Ledent et al. [20] | Human Vaccines & Immunotherapeutics | Funded by GlaxoSmithKline Biologicals SA, NCT01890447 | No |
| Liao et al. [21] | Vaccine | Grant from the Health Medical Research Funding, Food and Health Bureau, Government of Hong Kong (number 14130942, 2015) | No |
| Liao et al. [22] | Vaccine | Funded by the Health and Medical Research Fund of the Food and Health Bureau of the Hong Kong SAR Government (reference no. 16150852) | No |
| Lloyd et al. [23] | Patient Preference & Adherence | Conducted on behalf of Sanofi Pasteur MSD | No |
| Marshall et al. [24] | Vaccine | Funded by Novartis Vaccines | No |
| Ngorsuraches et al. [25] | Journal of Pharmaceutical Policy and Practice | No funding | No |
| Oteng et al. [26] | Sexually Transmitted Infections | Not reported | No |

| Study | Other | | |
| --- | --- | --- | --- |
|  | **Journal** | **Source of funding** | **Additional eligible studies** |
| Pereira et al. [27] | Vaccine | Grant from Becton, Dickinson & Company | No |
| Poulos et al. [28] | Vaccine | Funded by GlaxoSmithKline Biologicals SA, GSK study identifier: HO-14-13995 | No |
| Poulos et al. [29] | Value in Health | Funded by GlaxoSmithKline (producing MenHibrix) | No |
| Poulos et al. [30] | Vaccine | Funded by GlaxoSmithKline Biologicals S.A., GSK Study identifier: HO-13-14114 | No |
| Poulos et al. [31] | Social Science & Medicine | Funded by PATH and the Bill & Melinda Gates Foundation | No |
| Sadique et al. [32] | PLoS One | Grant-in-aid provided by the City Health Economics Centre, City University and the Health Protection Agency (London) | No |
| Sapède et al. [33] | International Journal of Market Research | Researcher employed by Aventis Pasteur | No |
| Seanehia et al. [34] | Vaccine | Funded by interdisciplinary research program PRINCEPS (Programme de recherche interdisciplinaire sur les crises et la protection sanitaires) at University of Sorbonne Paris Cité | No |
| Shono et al. [35] | Vaccine | Grant-in-Aid for Scientific Research (C) by Japan Society for the Promotion of Science (JSPS) KAKENHI Grant Number 25460817 | No |
| Shono et al. [36] | Human Vaccines & Immunotherapeutics | Grant-in-Aid for Scientific Research (C) from the Japan Society for the Promotion of Science (JSPS) KAKENHI (25460817) | No |
| Sun et al. [37] | Vaccine | Funded by the Fourth Round of Three-Year Public Health Action Plan of Shanghai, China (No. 15GWZK0101) | No |
| Veldwijk et al. [38] | Vaccine | Not reported | No |
| Verelst et al. [39] | Vaccine | Funded by the Global Minds initiative at the University of Antwerp | No |
| Verelst et al. [40] | Social Science & Medicine | Supported by the Antwerp Study Centre for Infectious Diseases (ASCID) at the University of Antwerp  Researchers supported by the Research Foundation Flanders (project no. G043815N) and postdoctoral fellowship | No |
| Wang et al. [41] | PLoS One | Funded by the Channel 7 Research Foundation (Project Reference No. 14897) | No |
| Wong et al. [42] | Value in Health | Funded by the Health and Medical Research Fund, Food and Health Bureau, Hong Kong SAR (reference no. 131120652) | No |

**References**

1. Adams J, Bateman B, Becker F, Cresswell T, Flynn D, McNaughton R, et al. Effectiveness and acceptability of parental financial incentives and quasi-mandatory schemes for increasing uptake of vaccinations in preschool children: systematic review, qualitative study and discrete choice experiment. Health Technol Assess. 2015;19(94):1-176.

2. Arbiol J, Yabe M, Nomura H, Borja M, Gloriani N, Yoshida S. Using discrete choice modeling to evaluate the preferences and willingness to pay for leptospirosis vaccine. Hum Vaccin Immunother. 2015;11(4):1046-56.
3. Bishai D, Brice R, Girod I, Saleh A, Ehreth J. Conjoint analysis of french and german parents’ willingness to pay for meningococcal vaccine. Pharmacoeconomics. 2007;25(2):143-54.

4. Brown DS, Poulos C, Reed Johnson F, Chamiec-Case L, Messonnier ML. Adolescent girls’ preferences for HPV vaccines: A discrete choice experiment. Adv Health Econ Health Serv Res. 2014;24;93-121.
5. Brown DS, Reed Johnson F, Poulos C, Messonnier ML. Mothers’ preferences and willingness to pay for vaccinating daughters against human papillomavirus. Vaccine. 2010;28(7):1702-8.

6. de Bekker-Grob EW, Hofman R, Donkers B, van Ballegooijen M, Helmerhorst TJM, Raat H, et al. Girls’ preferences for HPV vaccination: A discrete choice experiment. Vaccine. 2010;28(41):6692-7.
7. de Bekker-Grob EW, Jorien Veldwijk J, Jonker M, Donkers B, Huisman J, Buis S, et al. The impact of vaccination and patient characteristics on influenza vaccination uptake of elderly people: A discrete choice experiment. Vaccine. 2018;36(11):1467-76.

8. Determann D, Korfage IJ, Fagerlin A, Steyerberg EW, Bliemer MC, Voeten HA, et al. Public preferences for vaccination programmes during pandemics caused by pathogens transmitted through respiratory droplets – a discrete choice experiment in four European countries, 2013. Euro Surveill. 2016;21(22):pii=30247.
9. Determann D, Korfage IJ, Lambooij MS, Bliemer M, Richardus JH, Steyerberg EW, et al. Acceptance of vaccinations in pandemic outbreaks: A discrete choice experiment. PLoS One. 2014;9(7):e102505.

10. Eilers R, de Melker HE, Veldwijk J, Krabbe PFM. Vaccine preferences and acceptance of older adults. Vaccine. 2017;35(21):2823-30.
11. Flood EM, Ryan KJ, Rousculp MD, Beusterien KM, Block SL, Hall MC, et al. A survey of children’s preferences for influenza vaccine attributes. Vaccine. 2011;29(26):4334-40.
12. Flood EM, Ryan KJ, Rousculp MD, Beusterien KM, Divino VM, Block SL, MD, et al. Parent preferences for pediatric influenza vaccine attributes. Clin Pediatr. 2011;50(4):338-47.
13. Gidengil C, Lieu TA, Payne K, Rusinak D, Messonnier M, Prosser LA. Parental and societal values for the risks and benefits of childhood combination vaccines. Vaccine. 2012;30(23):3445-52.
14. Guo N, Zhang G, Zhu D, Wang J, Shi L. The effects of convenience and quality on the demand for vaccination: Results from a discrete choice experiment. Vaccine. 2017;35(21):2848-54.
15. Hall J, Kenny P, King M, Louviere J, Viney R, Yeoh A. Using stated preference discrete choice modelling to evaluate the introduction of varicella vaccination. Health Econ. 2002;11(5):457-65.
16. Hofman R, de Bekker-Grob EW, Raat H, Helmerhorst TJM , van Ballegooijen M, Korfage IJ. Parents’ preferences for vaccinating daughters against human papillomavirus in the Netherlands: A discrete choice experiment. BMC Public Health. 2014;14:454.
17. Hofman R, de Bekker-Grob EW, Richardus JH, de Koning HJ, van Ballegooijen M, Korfage IJ. Have preferences of girls changed almost 3 years after the much debated start of the HPV vaccination program in the Netherlands? A discrete choice experiment. PloS One. 2014;9(8):e104772.
18. Huang Z, Wagner AL, Lin M, Sun X, Zikmund-Fisher BJ, Boulton ML, et al. Preferences for vaccination program attributes among parents of young infants in Shanghai, China. Hum Vaccin Immunother. 2020:1-6.

19. Lambooij MS, Harmsen IA, Veldwijk J, de Melker H, Mollema L, van Weert YWM. Consistency between stated and revealed preferences: A discrete choice experiment and a behavioural experiment on vaccination behaviour compared. BMC Med Res Methodol. 2015;15:19.

20. Ledent E, Gabutti G, de Bekker-Grob EW, Alcázar Zambrano JL, Campins Martí M, Del Hierro Gurruchaga MT, et al. Attributes influencing parental decision-making to receive the Tdap vaccine to reduce the risk of pertussis transmission to their newborn – outcome of a cross-sectional conjoint experiment in Spain and Italy. Hum Vaccin Immunother. 2019;15(5):1080-91.

21. Liao Q, Lam WWT, Wong CKH, Lam C, Chen J, Fielding R. The relative effects of determinants on Chinese adults’ decision for influenza vaccination choice: What is the effect of priming? Vaccine. 2019;37(30):4124-32.

22.Liao Q, Ng TWY, Cowling BJ. What influenza vaccination programmes are preferred by healthcare personnel? A discrete choice experiment. Vaccine 2020;38(29):4557-62.

23.Lloyd AJ et al. What are the preferences of health care professionals in Germany regarding fully liquid, ready-to-use hexavalent pediatric vaccine versus hexavalent pediatric vaccine that needs reconstitution? Patient Prefer Adherence 2015;9:1517-24.

24. Marshall HS, Chen G, Clark M, Ratcliffe J. Adolescent, parent and societal preferences and willingness to pay for meningococcal B vaccine: A Discrete Choice Experiment. Vaccine. 2016;34(5):671-7.

25. Ngorsuraches S, Nawanukool K, Petcharamanee K, Poopantrakool U. Parents’ preferences and willingness- to-pay for human papilloma virus vaccines in Thailand. J Pharm Policy Pract. 2015;8(1):20.
26. Oteng B, Marra F, Lynd LD, Ogilvie G, Patrick D, Marra CA. Evaluating societal preferences for human papillomavirus vaccine and cervical smear test screening programme. Sex Transm Infect. 2011;87(1):52-7.
27. Pereira CCA, Mulligan M, Bridges JFP, Bishai D. Determinants of influenza vaccine purchasing decision in the US: A conjoint analysis. Vaccine. 2011;29(7):1443-7.

28. Poulos C, Curran D, Anastassopoulou A, de Moerlooze L. German travelers’ preferences for travel vaccines assessed by a discrete choice experiment. Vaccine. 2018;36(7):969-78.
29. Poulos C, Reed Johnson F, Krishnarajah G, Anonychuk A, Misurski D. Pediatricians’ preferences for infant meningococcal vaccination. Value Health. 2015;18(1):67-77.

30. Poulos C, Standaert B, Sloesen B, Stryjewska I, Janitsary A, Hauber B. Preferences for vaccines against children’s diarrheal illness among mothers in Poland and Hungary. Vaccine. 2018;36(40):6022-9.
31. Poulos C, Yang JC, Levin C, Van Minh H, Giang KB, Nguyen D. Mothers’ preferences and willingness to pay for HPV vaccines in Vinh Long Province, Vietnam. Soc Sci Med. 2011;73(2):226-34.

32. Sadique MZ, Devlin N, Edmunds WJ, Parkin D. The effect of perceived risks on the demand for vaccination: Results from a discrete choice experiment. PLoS One. 2013;8(2):e54149.

33. Sapède C, Girod I. Willingness of adults in Europe to pay for a new vaccine: The application of discrete choice-based conjoint analysis. Int J Mark Res 2002;44(4):463-76.

34. Seanehia J, Treibich C, Holmberg C, Müller-Nordhorn J, Casin V, Raude J. Quantifying population preferences around vaccination against severe but rare diseases: A conjoint analysis among French university students, 2016. Vaccine. 2017;35(20):2676-84.

35. Shono A, Kondo M. Mothers’ preferences regarding new combination vaccines for their children in Japan, 2014. Hum Vaccin Immunother. 2017;13(4):766-71.
36. Shono A, Kondo M. Parents’ preferences for seasonal influenza vaccine for their children in Japan. Vaccine. 2014;32(39):5071-6.

37. Sun X, Wagner AL, Ji J, Huang Z, Zikmund-Fisher BJ, Boulton ML. A conjoint analysis of stated vaccine preferences in Shanghai, China. Vaccine. 2020;38(6):1520-5.
38. Veldwijk J, Lambooij MS, Bruijning-Verhagen PCJ, Smit HA, de Wit GA. Parental preferences for rotavirus vaccination in young children: A discrete choice experiment. Vaccine. 2014;32(47):6277-83.

39. Verelst F, Kessels R, Delva W, Beutels P, Willem L. Drivers of vaccine decision-making in South Africa: A discrete choice experiment. Vaccine. 2019;37(15):2079-89.
40. Verelst F, Willem L, Kessels R, Beutels P. Individual decisions to vaccinate one's child or oneself: A discrete choice experiment rejecting free-riding motives. Soc Sci Med. 2018;207:106-16.

41. Wang B, Chen G, Ratcliffe J, Afzali HHA, Giles L, Marshall H. Adolescent values for immunisation programs in Australia: A discrete choice experiment. PLoS One. 2017;12(7): e0181073.
42. Wong CKH, Man KKC, Ip P, Kwan M, McGhee SM. Mothers’ preferences and willingness to pay for human papillomavirus vaccination for their daughters: A discrete choice experiment in Hong Kong. Value Health. 2018;21(5):622-9.
