# Supplementary material for: Vaccine preferences driving vaccine-decision making of different target groups: a systematic review of choice-based experiments
Source: BMC Infect Dis. 2021 Aug 28;21:879. doi: 10.1186/s12879-021-06398-9 (PMC8397865; doi:10.1186/s12879-021-06398-9)
Supplement: Supplementary file 3 — Additional file 3: Overview of attributes included in each category/domain. A table giving insight into the way attributes were grouped under categories and domains. [file 12879_2021_6398_MOESM3_ESM.docx]

**Additional file 3 – Overview of attributes included in each category/domain**

In the table below, an overview is provided about the attributes included in each category. As delineated in the main text, categories included one or more domain(s) (subgroups). These domains are outlined in Table 1 as well.

**Table 1 Attributes included in each domain**

| *Category & domain* | *Attribute* | | |
| --- | --- | --- | --- |
| **Outcome** |  |  |  |
| Protection duration | Duration: duration of protection | Duration of vaccine effectiveness | Vaccine protection |
|  | Duration of protection | Length of protection |  |
|  | Duration of protection/duration of effect | Protection duration |  |
| Vaccine effectiveness | Cervical cancer/genital warts risk reduction | Effectiveness: how well the vaccine protects against meningococcal disease | Protection against cervical cancer/genital warts |
|  | Coverage (infection and subtypes) | Indirect protection | Protection against hepatitis B |
|  | Degree of protection against cervical cancer | Number of cases of diarrheal illness prevented in children under 5 years | Vaccine effectiveness |
|  | Diseases targeting in one vaccine | Number of cases of disease, disability and death prevented over 5 y | Vaccine effectiveness in reducing cervical cancer risk/in decreasing risk of influenza infection |
|  | Effectiveness (of the vaccine) | Number of serogroups of the bacteria covered | (Vaccine) efficacy |
| Vaccine risk | Adverse event: type and probability of adverse events commonly experienced | Risk of adverse events (after vaccination) | Severe adverse event |
|  | Common side effects e.g. mild fever, little pain | Risk of fever > 100.4 after receiving each set of vaccines over 6 months | Severity of health consequences: Severity of VAAE |
|  | Contamination risk | Risk of getting fever after office visit | Side effects (of vaccination) |
|  | Frequency of (severe) side effects | (Risk of) mild side-effects | Sore arm |
|  | Potential for side effects | (Risk of) serious/severe side-effects | Vaccine adverse events: probability of mild vaccine adverse events (mild flu-like symptoms) |
|  | (Probability of) dosing errors | Risk of side effects | Vaccine safety |
|  | Probability of handling errors related to the number of preparation steps | Runny/stuffy nose | VRSE |
|  | Probability of occurrence: probability of VAAE | Safety of the vaccine |  |
| **Process** |  |  |  |
| Dosing and visits | Booster vaccine needed after 5 y | Number of injections added to the schedule | Timing of shots after birth and before 7 months |
|  | Extra HepB dose | Number of injections per visit/for one doctor visit | Times of vaccination for one winter season |
|  | Injections: number of injections (including the meningococcal B vaccine) that may occur at each visit | Number of shots per visit and total number of shots over 6 months | Vaccine schedule |
| *Category & domain* | *Attribute* | | |
| **Process** |  |  |  |
| Dosing and visits | Need for booster | Number of vaccine shots/given vaccinations/doses (of the vaccine) |  |
|  | Number of additional doctor visits required | Programme duration |  |
| Service delivery | Availability of appointments | Location of shot | Vaccination sites |
|  | Healthcare facility of vaccine administration | Vaccination arrangement procedure |  |
|  | Healthcare professional administering vaccinations and location of appointments | Vaccination location |  |
|  | Location for/of vaccination | Vaccination service hours |  |
| Target group | Target for protection | Which parents would receive a reward |  |
|  | Target group |  |  |
| Time | Number of months required for the vaccine | Speed | Waiting time at each appointment |
|  | Preparation steps | Vaccine will become active/absorption time |  |
|  | Preparation time | Waiting time at clinic |  |
| Vaccination age | Age at which protection begins |  |  |
|  | Age of/at vaccination |  |  |
| Vaccine accessibility | Accessibility |  |  |
|  | Distance |  |  |
| Vaccine administration | Injection avoided | Mode (of administration) |  |
|  | How vaccine is given | Vaccine delivery mechanism |  |
| Vaccine content | Mercury-containing preservative | Type of device |  |
|  | Thimerosal (free) | Virus type |  |
| **Cost** |  |  |  |
| Cost | Cost for all doses | Out-of-pocket price of vaccine | Total out-of-pocket costs to parents |
|  | Cost: cost of the vaccine course to you | Payment for one doctor visit | Type of parental reward |
|  | Cost per visit/person/vaccine | Parental reward value (received when full schedule of vaccinations is completed) | Vaccine cost |
|  | Cost per visit and total cost over 6 months | Personal cost of vaccines | (Vaccine) costs for 3 doses of (HPV) vaccine |
|  | Out-of-pocket cost (of the vaccination) | Price (of hypothetical vaccine/vaccination) | Vaccination price for one winter season |
| **Other** |  |  |  |
| Context | Attitude of social environment: number of friends getting their child vaccinated | Local coverage |  |
|  | Community vaccination coverage rate | Number vaccinated |  |
|  | Epidemic context | Population coverage |  |
|  | Immunization coverage at age 2 years in respondent's community | Proportion of colleagues intending to take SIV |  |

| *Category & domain* | *Attribute* | | |
| --- | --- | --- | --- |
| **Other** |  |  |  |
| Disease risk | Burden of disease | How common is the disease among children without vaccination | Probability of occurrence: probability of infection |
|  | Case-fatality ratio | Infection probability | Severity of disease/illnesses prevented by vaccine |
|  | Chance of contracting the disease without vaccination | Infection risk without vaccination | Severity of health consequences: severity of infection |
|  | Disease target | Lifetime cervical cancer/genital wart risk | Susceptibility |
|  | Health impacts of the disease | Mortality | Vaccination(s) & source(s) of infection |
| Information | How information about vaccination (benefits and risks) is provided prior to appointment | (Information on) vaccine coverage | Source of information that the vaccine causes problems: a child has been hospitalized after getting the vaccine |
|  | How information on reducing risk of your child getting diseases by having the vaccinations is provided prior to the appointment | Media coverage about the vaccine | Source of information that the vaccine is safe |
|  | Information | Social network, friends, Facebook, twitter | TV, newspaper and radio |
| Other | Experience of using this hexavalent vaccine device available on the German market | Storage |  |
|  | Pap smear frequency | Vaccine manufacturer |  |
|  | Possibility to choose for hepatitis B or not | Vaccine testing |  |
| Other disease related factors | Availability of curative treatment | Duration of illness-prevented by vaccine |  |
|  | Clinical symptoms | How disease is spread |  |
| Vaccine advice/support | Advice regarding the vaccine | Health authorities support |  |
|  | Doctors’ advice | Recommended by |  |

HepB = Hepatitis B; HPV = Human Papilloma Virus; SIV = Seasonal Influenza Vaccine; VAAE = Vaccine Associated Adverse Event; VRSE = Vaccine Related Side Effect; y = year(s)
