# Supplementary material for: Vaccine preferences driving vaccine-decision making of different target groups: a systematic review of choice-based experiments
Source: BMC Infect Dis. 2021 Aug 28;21:879. doi: 10.1186/s12879-021-06398-9 (PMC8397865; doi:10.1186/s12879-021-06398-9)
Supplement: Supplementary file 4 — Additional file 4: Quality assessment. A detailed description of the outcomes of the quality assessment performed. [file 12879_2021_6398_MOESM4_ESM.docx]

**Additional file 4 – Quality assessment**

An overview of the quality assessment of all 42 studies is presented in Table 6 of the main text. As previously indicated, quality scores ranged from 5.5 to 12.5, with an average score of 9.3. It was observed that quality scores have not improved over time, as average scores of studies published between 2000-5, 2006-10, 2011-5 and 2016-20 were 8.8, 10.5, 9.1 and 9.4 respectively. However, industry-funded studies scored lower than non-industry funded studies (mean of resp. 8.5 and 9.5). Four categories were distinguished and overall studies scored best on criteria targeting *analysis* (mean: 0.84), followed by *choice task design* and *conduct* (mean of resp. 0.70 and 0.65). The lowest score was administered on the *experimental design* (mean: 0.55).

With respect to the *choice task design*, the majority of the studies (59.5%) not involved the target population in the process of identifying attributes and levels. This may limit respondents’ comprehension of and engagement with choice tasks, particularly for omitted attributes [1]. In addition, conceptual overlap was observed to a greater or lesser extent in 25 of the 42 studies (59.5). This means that in more than half of the studies attributes addressed similar topics (domains) and not varied independently. In two studies, conceptual overlap even occurred in more than 50% of the included attributes. Nonetheless, attributes enclosed in thirty studies (71.4%) were uni-dimensional and not covered multiple aspects (e.g. number of visits and amount of injections per visit). As vaccination is not mandatory in most countries, 31 studies (73.8%) provided respondents an option to remain undecided (opt-out) at first or second instance.

As indicated in the main text, the category of the *experimental design* consisted of one criterion. The majority of the studies (63.4%) not fully satisfied this criterion and had a score below 1. Although a third of the studies (38.1%) used fractional factorial designs that showed elements of orthogonality, level balance and/or minimum overlap, only fifteen studies (35.7%) used fractional factorial designs that were optimal or efficient and estimated parameter effects with precision. According to Street & Burgess [2], the elements proposed by Huber & Zwerina [3] are not required to create efficient designs nor ensure optimality. Minimum overlap results for instance in optimal main effects only, while excluding estimation of interaction effects [1]. Among studies with efficient experimental designs, a commonly used measure to improve efficiency and accuracy of preferences was to maximize D-efficiency/optimality [1,4].

Varying scores were administered on the elements of *conduct*. Whereas the target population was in almost all studies appropriate for the research objective, response rates of individual studies were hardly sufficient to minimize response bias. In nineteen studies (45.2%) response rates were less than 25% or not reported and in fourteen studies (33.3%) between 25%-50%. Only nine studies (21.4%) reported a response rate of at least 75%. As low response rates may infer selection bias amongst respondents, SP captured in these DCEs may not be fully representative for the target population [5]. Controversially, sampling frames applied by more than half of the studies (22/42, 52.4%) was adequately targeting the entire target population and was representative.

In line with recommendations of Lancsar et al. [1], the validity of choice task designs and survey features were pilot tested in 81% of the studies (34/42). Nearly half of these studies (16/34) conducted a pilot among the target population to test for understanding, complexity and/or appropriateness of attributes and levels. In the remaining studies the target population was likely to be involved, but participants of the pilot were not clearly addressed.

The last category of the quality assessment includes the *analysis* of data. Almost all studies performed well on at least three of the four criteria covering analysis. Studies managed to choose econometric models that were appropriate for the choice task and number of alternatives (vaccine scenarios) presented. In addition, most studies (78.6%) used models that accounted for serial correlation of choices or added a random effects specification to avoid overestimation of differences in vaccine preference [1,5]. MXL/RPL were most often used to obtain parameter estimates for multinomial choice structures (see review study characteristics). As literature demonstrated that confounding occurs among utility scales underlying parameter estimates, these estimates could not be directly compared [1,5]. Instead, 35 studies (83.3%) used a common, comparable scale (metric) to interpret relative attribute effects. In accordance with recommendations of Louviere et al. [1,6], marginal rates of substitution, predicted probability analysis, WTP, best-worst attribute scaling and partial-log likelihood analysis were most commonly used as metric to highlight and compare preferences for vaccine attributes.

When computing scores on the four categories into a total score and comparing this to the pre-defined quality threshold, sixteen of the 42 studies passed the quality assessment and were regarded as high-quality studies. This means that less than half (38.1%) had a total score of at least 10 and were included in the data comparison. Total scores of the remaining studies (61.9%) varied between 5.5 and 9.5 and were considered insufficient to exclude most threats to validity. These were ‘lower-quality studies’ and were only included in the robustness analyses (and not in the primary data comparison).

**References**

1. Lancsar E, Louviere J: Conducting discrete choice experiments to inform healthcare decision making: A user’s guide. Pharm Econ. 2008;26(8):661–677.

2. Street DA, Burgess L, Louviere JJ. Quick and easy choice sets: constructing optimal and nearly optimal stated choice experiments. Int J Res Mark. 2005; 22(4):459-70.

3. Huber J, Zwerina K. The importance of utility balance in efficient choice designs. J Mark Res. 1996;33(3):307-17.

4. Reed Johnson F, et al. Constructing experimental designs for discrete-choice experiments: Report of the ISPOR conjoint analysis experimental design good research practices task force. Value Health. 2013;16(1):3- 13.

5. Mandeville KL, Lagarde M, Hanson K. The use of discrete choice experiments to inform health workforce policy: A systematic review. BMC Health Serv Res. 2014;14(1):367.

6. Lancsar E, Louviere J, Flynn T. Several methods to investigate relative attribute impact in stated preference experiments. Soc Sci Med. 2007;64(8):1738-53.
