# Supplementary material for: Vaccine preferences driving vaccine-decision making of different target groups: a systematic review of choice-based experiments
Source: BMC Infect Dis. 2021 Aug 28;21:879. doi: 10.1186/s12879-021-06398-9 (PMC8397865; doi:10.1186/s12879-021-06398-9)
Supplement: Supplementary file 5 — Additional file 5: Overview of vaccine attributes per study. A detailed overview of the vaccine attributes and domains used in each of the studies (incl. Statistical significance). [file 12879_2021_6398_MOESM5_ESM.docx]

**Additional file 5 – Overview of vaccine attributes per study**

The first table outlines the attributes and domains of each high-quality study (incl. statistical significance), while the second table reports the attribute information of lower-quality studies.

**Table 1. Overview of the vaccine attributes and domains used in each high-quality study**

| **Study** | **Target group** | **Vaccine attributes** | **Domains** | **Statistical significance** |
| --- | --- | --- | --- | --- |
| Adams et al. [1] | Representatives | 8 attributes, 2-6 levels  HC professional administering vaccinations and location of appointments, how info about vaccination (benefits and risks) is provided prior to appointment, how info on reducing risk of your child getting diseases by having the vaccinations is provided prior to the appointment, availability of appointments, type of parental reward, parental reward value (received when full schedule of vaccinations is completed), which parents would receive a reward, waiting time at each appointment | Service delivery (2x)  Information (2x)  Cost (2x)  Target group  Time | Statistical significance reported  Pooled data: target group, time, cost (cash), cost (value reward), service delivery (pharmacist) at p<0.01; service delivery (community nurse), information (charts/pictures) significant at p<0.05  Not statistically significant: information (format of info), service delivery (availability appointments) |
| Brown et al. [2] | Representatives | 4 attributes, 2-4 levels  Protection against cervical cancer, protection against genital warts, duration of protection, out-of-pocket cost | Vaccine effectiveness (2x)  Protection duration  Cost | Statistical significance reported  All attributes statistically significant for WTP and uptake  2x vaccine effectiveness, cost, protection duration (2, 10,lifetime) at p<0.01, except protection duration (5) |
| Brown et al. [3] | Vaccinees | 4 attributes, 2-4 levels  Protection against cervical cancer, protection against genital warts, duration of protection, out-of-pocket cost | Vaccine effectiveness (2x)  Protection duration  Cost | Statistical significance reported  Pooled data: vaccine effectiveness (50%), vaccine effectiveness (no warts), protection duration (2 yrs), cost significant at p<0.05 |
| de Bekker-Grob  et al. [4] | Vaccinees | 5 attributes, 3 levels  Protection against cervical cancer, protection duration, serious side-effects, mild side-effects, age of vaccination | Vaccine effectiveness  Protection duration  Vaccine risk (2x)  Vaccination age | Statistical significance reported (no exact p-values)  Vaccine effectiveness, protection duration, 2x vaccine risk, vaccination age significant at p<0.05 (all attributes) |
| de Bekker-Grob  et al. [5] | Vaccinees | 5 attributes, 2-4 levels  Effectiveness, risk of severe side effects, risk of mild side effects, protection duration, vaccine will become active/absorption time | Vaccine effectiveness  Vaccine risk (2x)  Protection duration  Time | Statistical significance reported (no exact p-values)  Vaccine effectiveness, 2x vaccine risk, protection duration statistically significant at p<0.01  Not statistically significant at p<0.01: time (p=0.25) |

| **Study** | **Target group** | **Vaccine attributes** | **Domains** | **Statistical significance** |
| --- | --- | --- | --- | --- |
| Determann et al. [6] | Vaccinees | 5 attributes, 2-6 levels, 2 scenarios  Effectiveness of vaccine, safety of the vaccine, advice regarding the vaccine, media coverage about the vaccine, out-of-pocket costs | Vaccine effectiveness  Vaccine risk  Vaccine advice/support Information  Cost | Statistical significance reported  Class 1: vaccine effectiveness, vaccine risk (no experience), vaccine advice/support (family discourage, doctor recommends, doctor discourages, government recommends, international recommends), information (traditional negative, 2x social media), cost at p<0.01  Class 2: vaccine effectiveness, vaccine risk (no experience), vaccine advice/support (family discourage, doctor recommends, doctor discourages, government recommends, international recommends), information (traditional negative, social medial negative), cost at p<0.01; information (positive) at p<0.05 |
| Determann et al. [7] | Vaccinees | 5 attributes, 2-6 levels, 2 scenarios  Effectiveness of the vaccine, safety of the vaccine, advice regarding the vaccine, media attention about the vaccine, out-of-pocket costs | Vaccine effectiveness  Vaccine risk  Vaccine advice/support  Information  Cost | Statistical significance reported  Class 1: vaccine effectiveness, vaccine risk, vaccine advice/support, information (except social media negative), cost significant at p<0.01  Class 2: vaccine effectiveness, vaccine risk, vaccine advice/support, information, cost significant at p<0.01 (all attributes)  Class 3: vaccine risk, vaccine advice (doctor recommends, doctor discourages, information (traditional media), cost at p<0.01; vaccine advice/support (family discourage, government/institutions recommend) at p<0.05; vaccine advice/support (international organization recommends), information (social media positive) at p<0.10  Not significant class 3: vaccine effectiveness |
| Hofman et al. [8] | Representatives | 4 attributes, 3 levels  Degree of protection against cervical cancer, duration of protection, risk of serious side-effects, age at vaccination | Vaccine effectiveness  Protection duration  Vaccine risk  Vaccination age | Statistical significance reported  Vaccine effectiveness, protection duration, vaccine risk and vaccination age significant at p<0.01 in table vs. p<0.05 in text (all attributes) |
| Hofman et al. [9] | Vaccinees | 5 attributes, 2-3 levels  Degree of protection against cervical cancer, duration of protection, risk of mild side-effects, age at vaccination, number of doses of the vaccine | Vaccine effectiveness  Protection duration  Vaccine risk  Vaccination age  Dosing and visits | Statistical significance reported  All 5 attributes statistically significant  Class 1: vaccine risk, vaccine effectiveness, protection duration (25 yrs) at p<0.01; protection duration (lifetime) at p<0.05, dosing and visits (3) at p<0.10  Not statistically significant: vaccination age  Class 2: vaccine risk, vaccine effectiveness, protection duration, vaccination age (14 yrs), dosing and visits at p<0.01  Class 3: vaccine risk, vaccine effectiveness, protection duration, vaccination age at p<0.01; dosing and visits at p<0.05 |

| **Study** | **Target group** | **Vaccine attributes** | **Domains** | **Statistical significance** |
| --- | --- | --- | --- | --- |
| Marshall et al. [10] | Vaccinees and representatives | 5 attributes, 3-4 levels  Effectiveness: how well the vaccine protects against meningococcal disease, duration: duration of protection, adverse event: type and probability of adverse events commonly experienced, injections: number of injections (including the meningococcal B vaccine) that may occur at each visit, cost: cost of the vaccine course to you | Vaccine effectiveness  Protection duration  Vaccine risk  Dosing and visits  Cost | Statistical significance reported  Adolescent (vaccinees): vaccine effectiveness, protection duration, cost, vaccine risk (higher fever, no), dosing and visits (1, 3, 4) statistically significant at p <0.01  Adult: vaccine effectiveness, protection duration, risk, cost, dosing and visits (1, 2, 4) statistically significant at p<0.01; dosing and visits (3) significant at p<0.05  Adult with children (representatives): vaccine effectiveness, protection duration, cost, vaccine risk (higher, slight, none), dosing and visits (1, 2 and 4) statistically significant at p<0.01; vaccine risk (local) significant at p<0.05  Adult without children (vaccinees): vaccine effectiveness (50%, 90%), protection duration, vaccine risk (high, slight, none), dosing and visits (1, 2 and 4), cost significant at p<0.01; vaccine effectiveness (70%), vaccine risk (local) significant at p<0.05 |
| Poulos et al. [11] | Vaccinees | 8 attributes, 2-3 levels  Chance of contracting the disease without vaccination, health impacts of the disease, how disease is spread, availability of curative treatment, duration of protection, number of doses, number of months required for the vaccine, cost for all doses | Disease risk (2x)  Other disease related factor (2x)  Protection duration  Dosing and visits  Time  Cost | Statistical significance reported  Pooled data: 2x disease risk and cost significant p<0.01  Not statistically significant (p<0.01): 2x other disease related factors, protection duration, dosing and visits, time |
| Poulos et al. [12] | Health advisors | 6 attributes, 2-4 attributes  Age at which protection begins, number of cases of disease, disability and death prevented over 5 y, number of injections added to the schedule, number of additional doctor visits required, booster vaccine needed after 5 y, total out-of-pocket costs to parents | Vaccination age  Vaccine effectiveness  Dosing and visits (3x)  Cost | Statistical significance reported, but NO threshold/ description of statistical significance  Vaccination age, vaccine effectiveness, 3 targeting dosing and visits, cost statistically significant (all attributes) |
| Poulos et al. [13] | Representatives | 6 attributes, 2-4 levels  Number of cases of diarrheal illness prevented in children under 5 years, severity of illnesses prevented by vaccine, duration of illness-prevented by vaccine, how vaccine is given, number of doses, personal cost of vaccines | Vaccine effectiveness  Disease risk  Other disease related factor  Vaccine administration  Dosing and visits  Cost | Statistical significance not reported for individual attributes (only significance reported for preference differences between subgroups based on working status) |
| Shono et al. [14] | Representatives | 4 attributes, 4-5 levels  Diseases targeting in one vaccine, number of injections per doctor visit, payment for one doctor visit, risk of adverse events after vaccination | Vaccine effectiveness  Dosing and visits  Cost  Vaccine risk | Statistical significance not reported (p-values presented, no threshold)  Vaccine effectiveness, dosing and visits, cost, vaccine risk have p-value of p=0.000 (all attributes) |

| **Study** | **Target group** | **Vaccine attributes** | **Domains** | **Statistical significance** |
| --- | --- | --- | --- | --- |
| Veldwijk et al. [15] | Representatives | 5 attributes, 3 levels  Vaccine effectiveness, frequency of severe side effects, protection duration, healthcare facility of vaccine administration, out-of-pocket costs | Vaccine effectiveness  Vaccine risk  Protection duration  Service delivery  Cost | Statistical significance reported  4 out of 5 statistically significant  Vaccine effectiveness, vaccine risk (1 in 1,000,000, 1 in 10,000), protection duration, cost significant at p<0.001; vaccine risk (1 in 100,000) significant at p<0.10  Not statistically significant: service delivery |
| Verelst et al. [16] | Vaccinees and representatives | 6 attributes, 2-3 levels  Vaccine effectiveness, burden of disease, VRSE, accessibility, local coverage, population coverage | Vaccine effectiveness  Disease risk  Vaccine risk  Vaccine accessibility  Context (2x) | Statistical significance reported  Adult model (vaccinees): vaccine effectiveness, disease risk, vaccine risk, vaccine accessibility, 2 targeting context significant at p<0.05 (all attributes)  Child model (representatives): vaccine effectiveness, disease risk, vaccine risk, vaccine accessibility, 2 targeting context significant at p<0.05 (all attributes) |

VRSE = Vaccine Related Side Effect(s), y = year(s); Yrs = years.

*Lower-quality studies*

**Table 2. Overview of the vaccine attributes and domains used in each of the lower-quality studies**

| **Study** | **Target group** | **Vaccine attributes** | **Domains** | **Statistical significance** |
| --- | --- | --- | --- | --- |
| Arbiol et al. [17] | Vaccinees | 5 attributes, 3-6 levels  Efficacy, duration of protection, risk of side effects, number of vaccine shots, price | Vaccine effectiveness  Protection duration  Vaccine risk  Dosing & visits  Cost | Statistical significance reported  Pooled data: vaccine effectiveness, protection duration, vaccine risk, dosing and visits and cost significant at p<0.01 (all attributes) |
| Bishai et al. [18] | Representatives | 3 attributes, 2-5 levels  Number of serogroups of the bacteria covered, the duration of protection/duration of effect, price of hypothetical vaccine | Vaccine effectiveness  Protection duration  Cost | Statistical significance reported  France (pooled data of video/no video): cost, protection duration and vaccine effectiveness significant at p<0.01 (all attributes)  Germany (pooled data of video/no video): cost, vaccine effectiveness at significant at p<0.01  Not significant: protection duration |
| Eilers et al. [19] | Vaccinees | 6 attributes, 2-4 levels  Clinical symptoms, susceptibility, mortality, vaccine effectiveness, side effects, number of given vaccinations | Other disease related factor  Disease risk (2x)  Vaccine effectiveness  Vaccine risk  Dosing & visits | Statistical significance reported  Pooled data: other disease related factor (pertussis, pneumococcal), disease risk (mortality), disease risk (susceptibility 1%, 100%), vaccine effectiveness at p<0.01; dosing and visits at p<0.05; other disease related factor (herpes) at p<0.10  Not statistically significant: vaccine risk |
| Flood et al. [20] | Vaccinees | 4 attributes, 2 levels  Efficacy, runny/stuffy nose, sore arm, mode | Vaccine effectiveness  Vaccine risk (2x)  Vaccine administration | Statistical significance not reported |
| Flood et al. [21] | Representatives | 6 attributes, 2-3 levels  Efficacy, runny/stuffy nose, sore arm, mode of administration, mercury-containing preservative, virus type | Vaccine effectiveness  Vaccine risk (2x)  Vaccine administration  Vaccine content (2x) | Statistical significance not reported |
| Gidengil et al. [22] | Representatives | 5 attributes, 2-4 levels  Number of shots per visit and total number of shots over 6 months, immunization coverage at age 2 years in respondent's community, extra HepB dose, risk of fever > 100.4 after receiving each set of vaccines over 6 months, cost per visit and total cost over 6 months | Dosing & visits (2x)  Context  Vaccine risk  Cost | Statistical significance reported  Vaccine risk, context, dosing and visits (no injections per visit), cost significant at p<0.001  Not statistically significant: dosing and visits (extra dose HepB avoided) |

| **Study** | **Target group** | **Vaccine attributes** | **Domains** | **Statistical significance** |
| --- | --- | --- | --- | --- |
| Guo et al. [23] | Vaccinees | 7 attributes, 3-4 levels  Side effects, protection duration, protection against hepatitis B, vaccine schedule, vaccination sites, distance, out-of-pocket cost | Vaccine risk  Protection duration  Vaccine effectiveness  Dosing & visits  Service delivery  Vaccine accessibility  Cost | Statistical significance reported  6 out of 7 attributes significant at p<0.05.  Vaccine risk (1/150,000, 1/50,000), protection duration (5, 20 yrs), vaccine effectiveness, cost significant at p<0.01; dosing and visits (3), service delivery (third) at p<0.05; vaccine risk (1/100,000) and protection duration 1 (10 yrs) at p<0.10  Not statistically significant: dosing and visits (second), vaccine accessibility |
| Hall et al. [24] | Representatives | 7 attributes, 2-4 levels  Risk of mild side effects, risk of severe side effects, vaccine effectiveness, number vaccinated, health authorities support, location for vaccination, price of vaccination | Vaccine risk (2x)  Vaccine effectiveness  Context  Vaccine advice/support  Service delivery  Cost | Statistical significance not reported |
| Huang et al. [25] | Representatives | 5 attributes, 3-4 levels  Waiting time at clinic, timing of shots after birth and before 7 months, cost per visit, number of injections per visit, location of shot | Time  Dosing & visits (2x)  Cost  Service delivery | Statistical significance not reported |
| Lambooij et al. [26] | Representatives | 6 attributes, 2-3 levels  Infection risk without vaccination, side effects of vaccination, possibility to choose for hepatitis B or not, source of information that the vaccine is safe, source of information that the vaccine causes problems: a child has been hospitalized after getting the vaccine, attitude of social environment: number of friends getting their child vaccinated | Disease risk  Vaccine risk  Other  Information (2x)  Context | Statistical significance reported  Disease risk, vaccine risk, other, 2 targeting information, context significant at p<0.01 (all attributes) |
| Ledent et al. [27] | Vaccinees | 8 attributes, 2-4 levels  Vaccination(s) & source(s) of infection, cost per person, vaccination location, vaccine protection, recommended by, information, TV, newspaper and radio, social network, friends, Facebook, twitter | Disease risk  Cost  Service delivery  Protection duration  Vaccine advice/support  Information (3x) | Statistical significance reported  Spain: disease risk, cost, service delivery, protection duration, vaccine advice/support, 3x information significant at p<0.05 (all attributes)  Italy: disease risk, cost, service delivery, protection duration, vaccine advice/support (paediatrician, paediatrician, health authority), 3x information significant at p<0.05  Not statistically significant at p<0.05 (Italy): vaccine adv/supp (health authority recommends) |

| **Study** | **Target group** | **Vaccine attributes** | **Domains** | **Statistical significance** |
| --- | --- | --- | --- | --- |
| Liao et al. [28] | Vaccinees | 7 attributes, 2-4 levels  Infection probability, case-fatality ratio, vaccine safety, vaccine efficacy, out-of-pocket cost of the vaccination, community vaccination coverage rate, doctors’ advice | Disease risk (2x)  Vaccine risk  Vaccine effectiveness  Cost  Context  Vaccine advice/support | Statistical significance reported  2 targeting disease risk, vaccine risk, vaccine effectiveness, cost, context, vaccine advice/support significant at p<0.001 (all attributes) |
| Liao et al. [29] | Health advisors | 7 attributes, 2-4 levels  Vaccine efficacy, vaccine adverse events (probability of mild vaccine adverse events (mild flu-like symptoms)), programme duration, vaccination location, vaccination arrangement procedure, vaccination service hours, proportion of colleagues intending to take SIV | Vaccine effectiveness  Vaccine risk  Dosing & visits  Service delivery (3x)  Context | Statistical significance reported  Vaccine effectiveness, vaccine risk, 3x service delivery, context significant at p<0.05  Not statistically significant at p<0.05: dosing & visits |
| Lloyd et al. [30] | Health advisors | 5 attributes, 2-3 levels  Type of device, experience of using this hexavalent vaccine device available on the German market, preparation time, probability of handling errors related to the number of preparation steps, probability of dosing errors | Vaccine content  Other  Time  Vaccine risk (2x) | Statistical significance reported  Pooled data: vaccine content, other, time, vaccine risk 2x significant at p<0.001 (all attributes) |
| Ngorsuraches et al. [31] | Representatives | 4 attributes, 3 or 4 levels  Cervical cancer risk reduction, genital warts risk reduction, common side effects e.g. mild fever, little pain costs for 3 doses of HPV vaccine | Vaccine effectiveness (2x)  Vaccine risk  Cost | Statistical significance reported  Pooled data: 2x vaccine effectiveness, vaccine risk, cost significant at p<0.0001 (all attributes) |
| Oteng et al. [32] | Representatives | 7 attributes, 3-4 levels  Lifetime cervical cancer risk, pap smear frequency, lifetime genital wart risk, need for booster, target group, frequency of side effects, vaccine cost | Disease risk (2x)  Other  Dosing & visits  Target group  Vaccine risk  Cost | Statistical significance reported  Dosing and visits (5 yr, never), other (3 yrs), target group, vaccine risk, cost, 2x disease risk significant at p<0.05  Not statistically significant at p<0.05: dosing and visits (booster 10 yrs), other (smear test yearly, 5 yrs, never) |
| Pereira et al. [33] | Health advisors | 7 attributes, 2 levels  Speed, thimerosal, contamination risk, storage, preparation steps, dosing errors, price | Time (2x)  Vaccine content  Vaccine risk (2x)  Other  Cost | Statistical significance reported  Pooled data: 2x time, vaccine content, 2x vaccine risk, other, cost significant at p<0.01 (all attributes) |
| Poulos et al. [34] | Representatives | 3 attributes, 3-4 levels  Vaccine effectiveness in reducing cervical cancer risk, duration of vaccine effectiveness, vaccine cost for 3 doses of vaccine | Vaccine effectiveness  Protection duration  Cost | Statistical significance reported  Cost, vaccine effectiveness, protection duration, except for protection duration (10 yrs) significant at p<0.05 in text, p<0.01 in table (all attributes) |

| **Study** | **Target group** | **Vaccine attributes** | **Domains** | **Statistical significance** |
| --- | --- | --- | --- | --- |
| Sadique et al. [35] | Representatives | 3 attributes, 3 levels  Out-of-pocket price of vaccine, probability of occurrence: probability of infection and probability of VAAE, severity of health consequences: severity of infection and severity of VAAE | Cost  Disease risk  Vaccine risk | Statistical significance reported  Baseline regression model: cost, disease risk (severity, probability infection), vaccine risk (severity VAAE) significant at p<0.05  Not statistically significant at p<0.05: vaccine risk (probability VAAE)  Extended regression model (baseline + regret): vaccine risk, disease risk (severity VAAE, infection) significant at p<0.05  Not statistically significant at p<0.05: cost, vaccine risk, disease risk (probability) |
| Sapède et al. [36] | Representatives | 3 attributes, 2-5 levels  Price (€), length of protection, coverage (infection and subtypes) | Cost  Protection duration  Vaccine effectiveness | Statistical significance not reported |
| Seanehia et al. [37] | Vaccinees | 4 attributes, 2-5 levels  Severe adverse event, epidemic context, indirect protection, (information on) vaccine coverage | Vaccine risk  Context  Vaccine effectiveness  Information | Significant contribution reported (p-values presented, no p-value threshold)  Pooled data: vaccine risk, context, vaccine effectiveness, information have p<0.001 (all attributes) |
| Shono et al. [38] | Representatives | 6 attributes, 2-7 levels  Vaccination price for one winter season, times of vaccination for one winter season, vaccine effectiveness in decreasing risk of influenza infection, risk of adverse events, injection avoided, thimerosal free | Cost  Dosing & visits  Vaccine effectiveness  Vaccine risk  Vaccine administration  Vaccine content | Statistical significance reported  Cost, dosing and visits, vaccine effectiveness, vaccine risk, vaccine content significant at p<0.05  Not statistically significant at p<0.05: vaccine administration |
| Sun et al. [39] | Representatives | 7 attributes, 2-4 levels  Cost per vaccine, risk of getting fever after office visit, vaccine manufacturer, vaccine testing, vaccine effectiveness, severity of disease, how common is the disease among children without vaccination | Cost  Vaccine risk  Other (2x)  Vaccine effectiveness  Disease risk (2x) | Statistical significance reported  Vaccine risk, 2x other, vaccine effectiveness, disease risk (severity of disease) statistically significant at p<0.05  Not statistically significant at p<0.05: cost, disease risk (disease prevalence) |
| Verelst et al. [40] | Vaccinees and representatives | 6 attributes, 2-3 levels  Vaccine effectiveness, burden of disease, VRSE, accessibility, local coverage, population coverage | Vaccine effectiveness  Disease risk  Vaccine risk  Vaccine accessibility  Context (2x) | Statistical significance reported  Adult model (vaccinees): vaccine effectiveness, disease risk, vaccine risk, vaccine accessibility, 2x context significant at p<0.05 (all attributes)  Child model (representatives): vaccine effectiveness, 2x context, disease risk, vaccine accessibility, vaccine risk significant at p<0.05 (all attributes) |

| **Study** | **Target group** | **Vaccine attributes** | **Domains** | **Statistical significance** |
| --- | --- | --- | --- | --- |
| Wang et al. [41] | Vaccinees | 6 attributes, 2-3 levels  Disease target, location of vaccination, potential for side effects, vaccine delivery mechanism, price, target for protection | Disease risk  Service delivery  Vaccine risk  Vaccine administration  Cost  Target group | Statistical significance not reported (p-values presented, no p-value threshold)  Disease risk, service delivery, vaccine risk, vaccine administration, cost have p<0.05;  Target group p=0.274 |
| Wong et al. [42] | Representatives | 4 attributes, 4 levels  Protection against cervical cancer, protection duration, side effects, out-of-pocket cost | Vaccine effectiveness  Protection duration  Vaccine risk  Cost | Statistical significance reported  Vaccine effectiveness, protection duration, vaccine risk, cost significant at p<0.001 (all attributes) |

HepB = Hepatitis B; HPV = Human Papilloma Virus; SIV = Seasonal Influenza Vaccine; VAAE = Vaccine Associated Adverse Event; VRSE = Vaccine Related Side Effect; yrs = years.

**References**

1. Adams J, Bateman B, Becker F, Cresswell T, Flynn D, McNaughton R, et al. Effectiveness and acceptability of parental financial incentives and quasi-mandatory schemes for increasing uptake of vaccinations in preschool children: Systematic review, qualitative study and discrete choice experiment. Health Technol Assess. 2015;19(94):1-176.

2. Brown DS, Poulos C, Reed Johnson F, Chamiec-Case L, Messonnier ML. Adolescent girls’ preferences for HPV vaccines: A discrete choice experiment. Adv Health Econ Health Serv Res. 2014;24;93-121.
3. Brown DS, Reed Johnson F, Poulos C, Messonnier ML. Mothers’ preferences and willingness to pay for vaccinating daughters against human papillomavirus. Vaccine. 2010;28(7):1702-8.

4. de Bekker-Grob EW, Hofman R, Donkers B, van Ballegooijen M, Helmerhorst TJM, Raat H, et al. Girls’ preferences for HPV vaccination: A discrete choice experiment. Vaccine. 2010;28(41):6692-7.
5. de Bekker-Grob EW, Jorien Veldwijk J, Jonker M, Donkers B, Huisman J, Buis S, et al. The impact of vaccination and patient characteristics on influenza vaccination uptake of elderly people: A discrete choice experiment. Vaccine. 2018;36(11):1467-76.

6. Determann D, Korfage IJ, Fagerlin A, Steyerberg EW, Bliemer MC, Voeten HA, et al. Public preferences for vaccination programmes during pandemics caused by pathogens transmitted through respiratory droplets – a discrete choice experiment in four European countries, 2013. Euro Surveill. 2016;21(22):pii=30247.
7. Determann D, Korfage IJ, Lambooij MS, Bliemer M, Richardus JH, Steyerberg EW, et al. Acceptance of vaccinations in pandemic outbreaks: A discrete choice experiment. PLoS One. 2014;9(7):e102505.

8. Hofman R, de Bekker-Grob EW, Raat H, Helmerhorst TJM , van Ballegooijen M, Korfage IJ. Parents’ preferences for vaccinating daughters against human papillomavirus in the Netherlands: A discrete choice experiment. BMC Public Health. 2014;14:454.
9. Hofman R, de Bekker-Grob EW, Richardus JH, de Koning HJ, van Ballegooijen M, Korfage IJ. Have preferences of girls changed almost 3 years after the much debated start of the HPV vaccination program in the Netherlands? A discrete choice experiment. PloS One. 2014;9(8):e104772.

10. Marshall HS, Chen G, Clark M, Ratcliffe J. Adolescent, parent and societal preferences and willingness to pay for meningococcal B vaccine: A discrete choice experiment. Vaccine. 2016;34(5):671-7.

11. Poulos C, Curran D, Anastassopoulou A, de Moerlooze L. German travelers’ preferences for travel vaccines assessed by a discrete choice experiment. Vaccine. 2018;36(7):969-78.
12. Poulos C, Reed Johnson F, Krishnarajah G, Anonychuk A, Misurski D. Pediatricians’ preferences for infant meningococcal vaccination. Value Health. 2015;18(1):67-77.

13. Poulos C, Standaert B, Sloesen B, Stryjewska I, Janitsary A, Hauber B. Preferences for vaccines against children’s diarrheal illness among mothers in Poland and Hungary. Vaccine. 2018;36(40):6022-9.

14. Shono A, Kondo M. Parents’ preferences for seasonal influenza vaccine for their children in Japan. Vaccine. 2014;32(39):5071-6.

15. Veldwijk J, Lambooij MS, Bruijning-Verhagen PCJ, Smit HA, de Wit GA. Parental preferences for rotavirus vaccination in young children: A discrete choice experiment. Vaccine. 2014;32(47):6277-83.

16. Verelst F, Willem L, Kessels R, Beutels P. Individual decisions to vaccinate one's child or oneself: A discrete choice experiment rejecting free-riding motives. Soc Sci Med. 2018;207:106-16.

17. Arbiol J, Yabe M, Nomura H, Borja M, Gloriani N, Yoshida S. Using discrete choice modeling to evaluate the preferences and willingness to pay for leptospirosis vaccine. Hum Vaccin Immunother. 2015;11(4):1046-56.

18. Bishai D, Brice R, Girod I, Saleh A, Ehreth J. Conjoint analysis of French and German parents’ willingness to pay for meningococcal vaccine. Pharmacoeconomics. 2007;25(2):143-54.

19. Eilers R, de Melker HE, Veldwijk J, Krabbe PFM. Vaccine preferences and acceptance of older adults. Vaccine. 2017;35(21):2823-30.

20. Flood EM, Ryan KJ, Rousculp MD, Beusterien KM, Block SL, Hall MC, et al. A survey of children’s preferences for influenza vaccine attributes. Vaccine. 2011;29(26):4334-40.
21. Flood EM, Ryan KJ, Rousculp MD, Beusterien KM, Divino VM, Block SL, MD, et al. Parent preferences for pediatric influenza vaccine attributes. Clin Pediatr. 2011;50(4):338-47.
22. Gidengil C, Lieu TA, Payne K, Rusinak D, Messonnier M, Prosser LA. Parental and societal values for the risks and benefits of childhood combination vaccines. Vaccine. 2012;30(23):3445-52.

23. Guo N, Zhang G, Zhu D, Wang J, Shi L. The effects of convenience and quality on the demand for vaccination: Results from a discrete choice experiment. Vaccine. 2017;35(21):2848-54.
24. Hall J, Kenny P, King M, Louviere J, Viney R, Yeoh A. Using stated preference discrete choice modelling to evaluate the introduction of varicella vaccination. Health Econ. 2002;11(5):457-65.

25. Huang Z, Wagner AL, Lin M, Sun X, Zikmund-Fisher BJ, Boulton ML, et al. Preferences for vaccination program attributes among parents of young infants in Shanghai, China. Hum Vaccin Immunother. 2020:1-6.

26. Lambooij MS, Harmsen IA, Veldwijk J, de Melker H, Mollema L, van Weert YWM. Consistency between stated and revealed preferences: A discrete choice experiment and a behavioural experiment on vaccination behaviour compared. BMC Med Res Methodol. 2015;15:19.

27. Ledent E, Gabutti G, de Bekker-Grob EW, Alcázar Zambrano JL, Campins Martí M, Del Hierro Gurruchaga MT, et al. Attributes influencing parental decision-making to receive the Tdap vaccine to reduce the risk of pertussis transmission to their newborn – outcome of a cross-sectional conjoint experiment in Spain and Italy. Hum Vaccin Immunother. 2019;15(5):1080-91.

28. Liao Q, Lam WWT, Wong CKH, Lam C, Chen J, Fielding R. The relative effects of determinants on Chinese adults’ decision for influenza vaccination choice: What is the effect of priming? Vaccine. 2019;37(30):4124-32.

29. Liao Q, Ng TWY, Cowling BJ. What influenza vaccination programmes are preferred by healthcare personnel? A discrete choice experiment. Vaccine 2020;38(29):4557-62.

30. Lloyd AJ et al. What are the preferences of health care professionals in Germany regarding fully liquid, ready-to-use hexavalent pediatric vaccine versus hexavalent pediatric vaccine that needs reconstitution? Patient Prefer Adherence 2015;9:1517-24.

31. Ngorsuraches S, Nawanukool K, Petcharamanee K, Poopantrakool U. Parents’ preferences and willingness- to-pay for human papilloma virus vaccines in Thailand. J Pharm Policy Pract. 2015;8(1):20.
32. Oteng B, Marra F, Lynd LD, Ogilvie G, Patrick D, Marra CA. Evaluating societal preferences for human papillomavirus vaccine and cervical smear test screening programme. Sex Transm Infect. 2011;87(1):52-7.
33. Pereira CCA, Mulligan M, Bridges JFP, Bishai D. Determinants of influenza vaccine purchasing decision in the US: A conjoint analysis. Vaccine. 2011;29(7):1443-7.

34. Poulos C, Yang JC, Levin C, Van Minh H, Giang KB, Nguyen D. Mothers’ preferences and willingness to pay for HPV vaccines in Vinh Long Province, Vietnam. Soc Sci Med. 2011;73(2):226-34.

35. Sadique MZ, Devlin N, Edmunds WJ, Parkin D. The effect of perceived risks on the demand for vaccination: Results from a discrete choice experiment. PLoS One. 2013;8(2):e54149.

36. Sapède C, Girod I. Willingness of adults in Europe to pay for a new vaccine: The application of discrete choice-based conjoint analysis. Int J Mark Res 2002;44(4):463-76.

37. Seanehia J, Treibich C, Holmberg C, Müller-Nordhorn J, Casin V, Raude J. Quantifying population preferences around vaccination against severe but rare diseases: A conjoint analysis among French university students, 2016. Vaccine. 2017;35(20):2676-84.

38. Shono A, Kondo M. Mothers’ preferences regarding new combination vaccines for their children in Japan, 2014. Hum Vaccin Immunother. 2017;13(4):766-71.

39. Sun X, Wagner AL, Ji J, Huang Z, Zikmund-Fisher BJ, Boulton ML. A conjoint analysis of stated vaccine preferences in Shanghai, China. Vaccine. 2020;38(6):1520-5.

40. Verelst F, Kessels R, Delva W, Beutels P, Willem L. Drivers of vaccine decision-making in South Africa: A discrete choice experiment. Vaccine. 2019;37(15):2079-89.

41. Wang B, Chen G, Ratcliffe J, Afzali HHA, Giles L, Marshall H. Adolescent values for immunisation programs in Australia: A discrete choice experiment. PLoS One. 2017;12(7): e0181073.
42. Wong CKH, Man KKC, Ip P, Kwan M, McGhee SM. Mothers’ preferences and willingness to pay for human papillomavirus vaccination for their daughters: A discrete choice experiment in Hong Kong. Value Health. 2018;21(5):622-9.
