# Supplementary material for: Vaccine preferences driving vaccine-decision making of different target groups: a systematic review of choice-based experiments
Source: BMC Infect Dis. 2021 Aug 28;21:879. doi: 10.1186/s12879-021-06398-9 (PMC8397865; doi:10.1186/s12879-021-06398-9)
Supplement: Supplementary file 6 — Additional file 6: Importance rankings vaccine attributes. Tables outlining the frequency in which domains were reported among high- and lower-quality studies. [file 12879_2021_6398_MOESM6_ESM.docx]

**Additional file 6 – Importance rankings vaccine attributes**

The frequency in which domains were reported among high-quality studies is outlined in the first table and for all studies in the second table. Importance rankings were derived (second column) from the frequency of reporting (first column), with higher rankings for more frequently reported domains. If domains were not reported ‘-‘ is used.

**Table 1 Frequency of domains in high-quality studies**

| **Category** | *Vaccinees* | | *Representatives* | | *Pooled* |  |
| --- | --- | --- | --- | --- | --- | --- |
| Domain | **Number of studies (%)*** | **Rank** | **Number of studies (%)*** | **Rank** | **Number of studies (%)*** | **Rank** |
| **Outcome** |  |  |  |  |  |  |
| Protection duration | 6 (12.5) | 3 | 4 (8.3) | 5 | 10 (10.4) | 4 |
| Vaccine effectiveness | 9 (18.8) | 1 | 9 (18.8) | 1 | 18 (18.8) | 1 |
| Vaccine risk | 9 (18.8) | 1 | 5 (10.4) | 4 | 14 (14.6) | 2 |
| **Process** |  |  |  |  |  |  |
| Dosing & visits | 3 (6.3) | 5 | 6 (12.5) | 3 | 9 (9.4) | 5 |
| Service delivery | - | - | 3 (6.3) | 6 | 3 (3.1) | 10 |
| Target group | - | - | 1 (2.1) | 11 | 1 (1) | 15 |
| Time | 2 (4.2) | 7 | 1 (2.1) | 11 | 3 (3.1) | 10 |
| Vaccination age | 2 (4.2) | 7 | 2 (4.2) | 7 | 4 (4.2) | 7 |
| Vaccine accessibility | 1 (2.1) | 13 | 1 (2.1) | 11 | 2 (2.1) | 13 |
| Vaccine administration | - | - | 1 (2.1) | 11 | 1 (1) | 15 |
| **Cost** |  |  |  |  |  |  |
| Cost | 5 (10.4) | 4 | 8 (16.7) | 2 | 13 (13.5) | 3 |
| **Other** |  |  |  |  |  |  |
| Context | 2 (4.2) | 7 | 2 (4.2) | 7 | 4 (4.2) | 7 |
| Disease risk | 3 (6.3) | 5 | 2 (4.2) | 7 | 5 (5.2) | 6 |
| Information | 2 (4.2) | 7 | 2 (4.2) | 7 | 4 (4.2) | 7 |
| Other disease related factors | 2 (4.2) | 7 | 1 (2.1) | 11 | 3 (3.1) | 10 |
| Vaccine advice/support | 2 (4.2) | 7 | - | - | 2 (2.1) | 13 |
| Total | 48 |  | 48 |  | 96 |  |

*Note: due to rounding off the percentages, totals may not count up to 100%.

**Table 2 Frequency of domains high- and lower-quality studies combined**

| **Category** | *Vaccinees* | | *Representatives* | | *Pooled* |  |
| --- | --- | --- | --- | --- | --- | --- |
| Domain | **Number of studies (%)*** | **Rank** | **Number of studies (%)*** | **Rank** | **Number of studies (%)*** | **Rank** |
| **Outcome** |  |  |  |  |  |  |
| Protection duration | 9 (8.9) | 5 | 8 (5.6) | 6 | 17 (7.0) | 6 |
| Vaccine effectiveness | 16 (15.8) | 2 | 21 (14.8) | 2 | 37 (15.2) | 2 |
| Vaccine risk | 18 (17.8) | 1 | 23 (16.2) | 1 | 41 (16.9) | 1 |
| **Process** |  |  |  |  |  |  |
| Dosing & visits | 6 (5.9) | 6 | 13 (9.2) | 4 | 19 (7.8) | 4 |
| Service delivery | 3 (3.0) | 10 | 8 (5.6) | 6 | 11 (4.5) | 8 |
| Target group | 1 (1.0) | 16 | 2 (1.4) | 14 | 3 (1.2) | 18 |
| Time | 2 (2.0) | 13 | 5 (3.5) | 10 | 7 (2.9) | 10 |
| Vaccination age | 2 (2.0) | 13 | 2 (1.4) | 14 | 4 (1.6) | 16 |
| Vaccine accessibility | 3 (3.0) | 10 | 2 (1.4) | 14 | 5 (2.1) | 12 |
| Vaccine administration | 2 (2.0) | 13 | 3 (2.1) | 13 | 5 (2.1) | 12 |
| Vaccine content | - | - | 5 (3.5) | 10 | 5 (2.1) | 12 |
| **Cost** |  |  |  |  |  |  |
| Cost | 10 (9.9) | 3 | 21 (14.8) | 2 | 31 (12.8) | 3 |
| **Other** |  |  |  |  |  |  |
| Context | 6 (5.9) | 6 | 8 (5.6) | 6 | 14 (5.8) | 7 |
| Disease risk | 10 (9.9) | 3 | 9 (6.3) | 5 | 19 (7.8) | 4 |
| Information | 6 (5.9) | 6 | 4 (2.8) | 12 | 10 (4.1) | 9 |
| Other | - | - | 6 (4.2) | 9 | 6 (2.5) | 11 |
| Other disease related factors | 3 (3.0) | 10 | 1 (0.7) | 17 | 4 (1.6) | 16 |
| Vaccine advice/support | 4 (4.0) | 9 | 1 (0.7) | 17 | 5 (2.5) | 12 |
| Total | 101 |  | 142 |  | 243 |  |

*Note: due to rounding off the percentages, totals may not count up to 100%.
