# Supplementary material for: Vaccine preferences driving vaccine-decision making of different target groups: a systematic review of choice-based experiments
Source: BMC Infect Dis. 2021 Aug 28;21:879. doi: 10.1186/s12879-021-06398-9 (PMC8397865; doi:10.1186/s12879-021-06398-9)
Supplement: Supplementary file 7 — Additional file 7: Relative statistical significance (overall preferences). Tables giving insight into the amount of times domains were reported statistically significant in all studies (overall preferences). [file 12879_2021_6398_MOESM7_ESM.docx]

**Additional file 7 – Relative statistical significance (overall preferences)**

These tables give insight into the frequency of reporting relative statistical significance for included domains among studies capturing preferences of vaccinees and representatives (overall preferences). The first table covers the high-quality studies and the second includes lower-quality studies as well.

**Table 1 Statistical significance domains among high-quality studies (overall preferences)**

| Category & domain (n=)* | Statistical significance** | | | | | |
| --- | --- | --- | --- | --- | --- | --- |
|  | **P<0.10** | **P<0.05** | **P<0.01** | **P<0.001** | **Total** | **Not significant** |
| **Outcome** |  |  |  |  |  |  |
| Protection duration (n=10) | 0 | 4 | 7 | 1 | 12 | 1 |
| Vaccine effectiveness (n=14) | 0 | 8 | 12 | 1 | 21 | 1 |
| Vaccine risk (n=12) | 1 | 8 | 11 | 0 | 19 | 0 |
| **Process** |  |  |  |  |  |  |
| Dosing & visits (n=5) | 1 | 2 | 4 | 0 | 7 | 1 |
| Service delivery (n=2) | 0 | 1 | 0 | 0 | 1 | 2 |
| Target group (n=1) | 0 | 0 | 1 | 0 | 1 | 0 |
| Time (n=3) | 0 | 0 | 1 | 0 | 1 | 0 |
| Vaccination age (n=3) | 0 | 2 | 2 | 0 | 4 | 1 |
| Vaccine accessibility (n=2) | 0 | 2 | 0 | 0 | 2 | 2 |
| **Cost** |  |  |  |  |  |  |
| Cost (n=10) | 0 | 2 | 12 | 1 | 15 | 0 |
| **Other** |  |  |  |  |  |  |
| Context (n=2) | 0 | 4 | 0 | 0 | 4 | 0 |
| Disease risk (n=3) | 0 | 2 | 2 | 0 | 4 | 0 |
| Information (n=3) | 1 | 1 | 4 | 0 | 6 | 1 |
| Other dis. rel. factors (n=1) | 0 | 0 | 0 | 0 | 0 | 2 |
| Vaccine advice/support (n=2) | 1 | 0 | 4 | 0 | 5 | 0 |

*n=number of studies reporting a particular domain. 16/18 studies reported statistical significance (incl. legend) and/or p-values; ** *Information is based on main models and pooled data when available (if not, data of separate models/classes was used). Some studies included more than one attribute related to a particular domain. Totals could hence exceed the total number of studies incorporated.*

**Table 2 Statistical significance domains among high-and lower-quality studies (overall preferences)**

| **Category** | Statistical significance** | | | | | | | |
| --- | --- | --- | --- | --- | --- | --- | --- | --- |
| Domain (n=)* | **P<0.10** | **P<0.05** | **P<0.01** | **P<0.001** | **P<0.0001** | **Tot.** | **Not significant** |  |
| **Outcome** |  |  |  |  |  |  |  |  |
| Protection dur. (n=16) | 1 | 7 | 9 | 2 | 0 | 19 | 2 |  |
| Vaccine effect. (n=28) | 0 | 15 | 17 | 3 | 1 | 36 | 1 |  |
| Vaccine risk (n=31) | 2 | 17 | 15 | 5 | 1 | 39 | 2 |  |
| **Process** |  |  |  |  |  |  |  |  |
| Dosing & visits (n=11) | 1 | 6 | 5 | 1 | 0 | 13 | 3 |  |
| Service delivery (n=6) | 0 | 8 | 0 | 0 | 0 | 8 | 2 |  |
| Target group (n=3) | 0 | 1 | 1 | 0 | 0 | 2 | 1 |  |
| Time (n=4) | 0 | 0 | 3 | 1 | 0 | 4 | 2 |  |
| Vaccination age (n=2) | 0 | 2 | 2 | 0 | 0 | 4 | 1 |  |
| Vaccine access. (n=5) | 0 | 4 | 0 | 0 | 0 | 4 | 1 |  |
| Vaccine adm. (n=2) | 0 | 1 | 0 | 0 | 0 | 1 | 1 |  |
| Vaccine content (n=3) | 0 | 1 | 1 | 1 | 0 | 3 | 0 |  |
| **Cost** |  |  |  |  |  |  |  |  |
| Cost (n=25) | 0 | 9 | 17 | 4 | 1 | 31 | 1 |  |
| **Other** |  |  |  |  |  |  |  |  |
| Context (n=9) | 0 | 10 | 1 | 2 | 0 | 13 | 0 |  |
| Disease risk (n=13) | 0 | 12 | 5 | 2 | 0 | 19 | 1 |  |
| Information (n=6) | 1 | 8 | 6 | 0 | 0 | 15 | 1 |  |
| Other (n=5) | 0 | 3 | 2 | 1 | 0 | 6 | 0 |  |
| Other dis.rel. fact. (n=2) | 1 | 0 | 0 | 0 | 0 | 1 | 2 |  |
| Vaccine adv./supp. (n=4) | 1 | 2 | 4 | 1 | 0 | 8 | 0 |  |

*n=the number of studies reporting a particular domain. 38/45 studies reported statistical significance and/or attribute p-values; ***Information is based on main models and pooled data when available (if not, data of separate models/classes was used). Some studies included more than one attribute related to a particular domain. Totals could hence exceed the total number of studies incorporated.*
