# Supplementary material for: Vaccine preferences driving vaccine-decision making of different target groups: a systematic review of choice-based experiments
Source: BMC Infect Dis. 2021 Aug 28;21:879. doi: 10.1186/s12879-021-06398-9 (PMC8397865; doi:10.1186/s12879-021-06398-9)
Supplement: Supplementary file 8 — Additional file 8: Comparison of high- and lower-quality studies. A more detailed description of findings of the robustness analysis. [file 12879_2021_6398_MOESM8_ESM.docx]

**Additional file 8 – Comparison of high- and lower-quality studies**

When combining high- and lower-quality studies, eight additional studies focused on vaccinees, fourteen on representatives and three on health advisors. Vaccinees as well as representatives were targeted in one lower-quality study. In line with the approaches used for comparing high-quality studies, studies targeting health advisors were added to the representatives and outcomes of the study of Verelst et al. [1] were split (and grouped under vaccinees as well as representatives). Consequently, data of eighteen studies were compared for vaccinees and 27 for representatives. A complete overview of attribute information for high -and lower-quality studies is outlined in Additional file 5.

*Vaccinees*

Studies targeting vaccinees distinguished 101 attributes along sixteen domains (Figure 1). Overall, 42.6%, 18.8%, 9.9% and 28.7% of the attributes were classified as outcome, process, cost and other, respectively. Compared to high-quality studies, three additional domains were identified: service delivery, vaccine administration, vaccine advice/support. These were classified as process and other. Five of the six domains reported in high-quality studies were also incorporated in the overall top five (Additional file 6). Only dosing & visits (process) was excluded as it decreased from 6.3% to 5.9%, towards rank 6. With regard to the outcome measures, vaccine effectiveness and vaccine risk switched places, with vaccine risk now being most frequently reported. A remarkable change was the drop of the domain protection duration, which was previously reported in 12.5% of the studies and now decreased to 8.9% (from rank 3 to rank 5). Least reported domains were more or less the same among high- and lower-quality studies.

Sixteen studies targeting vaccinees reported relative statistical significance of attributes. Wang, Chen, Ratcliffe, Afzali, Giles & Marshall [2] not assessed statistical significance but provided p-values based on which it could be determined (using p<0.05). As shown in Table 1 of this file, outcome measures vaccine risk and vaccine effectiveness were most often statistically significant, which is in accordance with high-quality studies. The only inconsistency concerned disease risk (other). Whereas two high-quality studies reported it to be statistically significant (at p<0.01 and p<0.05), five lower-quality studies did so (p<0.001-p<0.05). This discrepancy could potentially be explained by the larger amount of lower-quality studies that incorporated one (or more) attribute(s) related to the risk of a disease (Figure 1; number of studies<total in Table 1). In line with high-quality studies, time was not statistically significant in any of the studies. By incorporating lower-quality studies the domain target group was also considered to be insignificant.

**Table 1 Overview of all studies reporting relative statistical significance of domains (vaccinees)**

| Category & domain (n=)* | Statistical significance** | | | | | |
| --- | --- | --- | --- | --- | --- | --- |
|  | **P<0.10** | **P<0.05** | **P<0.01** | **P<0.001** | **Total** | **Not significant** |
| **Outcome** |  |  |  |  |  |  |
| Protection duration (n=9) | 1 | 5 | 6 | 0 | 12 | 1 |
| Vaccine effectiveness (n=14) | 0 | 7 | 12 | 1 | 20 | 1 |
| Vaccine risk (n=14) | 1 | 7 | 12 | 1 | 21 | 1 |
| **Process** |  |  |  |  |  |  |
| Dosing & visits (n=6) | 1 | 3 | 3 | 0 | 7 | 1 |
| Service delivery (n=3) | 0 | 4 | 0 | 0 | 4 | 0 |
| Target group (n=1) | 0 | 0 | 0 | 0 | 0 | 1 |
| Time (n=2) | 0 | 0 | 0 | 0 | 0 | 2 |
| Vaccination age (n=2) | 0 | 1 | 2 | 0 | 3 | 1 |
| Vaccine accessibility (n=3) | 0 | 2 | 0 | 0 | 2 | 1 |
| Vaccine administration (n=1) | 0 | 1 | 0 | 0 | 1 | 0 |
| **Cost** |  |  |  |  |  |  |
| Cost (n=10) | 0 | 4 | 4 | 1 | 9 | 0 |
| **Other** |  |  |  |  |  |  |
| Context (n=4) | 0 | 5 | 0 | 1 | 6 | 0 |
| Disease risk (n=7) | 0 | 5 | 4 | 2 | 11 | 0 |
| Information (n=4) | 1 | 8 | 0 | 0 | 9 | 0 |
| Other dis. rel. factors (n=2) | 1 | 0 | 0 | 0 | 1 | 2 |
| Vaccine advice/support (n=4) | 1 | 2 | 4 | 1 | 8 | 0 |

*n=number of studies reporting domains. 17/18 studies reported statistical significance and/or attribute p-values; ** *Information is based on main models and pooled data when available (if not, data of separate models/classes was used). Some studies included more than one attribute related to a particular domain.*

*Totals could hence exceed the total number of studies incorporated.*

*Representatives*

A total of 142 attributes were used among all studies capturing preferences of representatives. Of these attributes, 36.6% were classified as outcome, 28.2% as process, 14.8% as cost and 20.4% as other. Attributes covered eighteen domains, implying that three domains were added. These domains were ‘vaccine advice/support’, ‘vaccine content’ and ‘other’. The latter included attributes that could not be grouped under the other seventeen domains (e.g. vaccine testing). Four of the five most frequently reported domains were in line with high-quality studies (Figure 1). Despite this correspondence at first glance, the inclusion of lower-quality studies changed the sequence and proportion of studies reporting particular domains. Vaccine effectiveness decreased for instance with 4.0% and was not most commonly reported anymore (i.e. dropped from rank 1 to 2). Instead another outcome-related measure, vaccine risk, was

most often used. The proportion of studies reporting this domain increased with more than 50%, which resulted in a rise from rank 4 to rank 1 (Additional file 6). This shift was partly due to conceptual overlap among attributes, as reported in the validity assessment. To illustrate this, four lower-quality studies included more than one risk-related attribute, while none high-quality study did so. In addition, risk-related attributes in lower-quality studies encompassed the risk of side effects as well as risks of dosing and handling. With respect to vaccine effectiveness, lower-quality studies also broadened the range of definitions and defined vaccine effectiveness for instance as ‘the number of serogroups of the bacteria covered’ and ‘cervical cancer risk reduction’. High-quality studies mainly adhered to ‘vaccine effectiveness’ or ‘efficacy’. Despite these inconsistencies between high- and lower-quality studies, the least reported domains were generally comparable between both.

In total, 21 of the 27 studies reported attributes’ relative statistical significance or outlined p-values based on which it could be determined (using p<0.05). Five alphas were used, with the strictest set at p<0.0001. In line with high-quality studies, outcome and cost-related measures were most commonly statistically significant (Table 2). The most obvious change introduced by the inclusion of lower-quality studies is the increase in frequency of reporting statistical significance for disease risk (other). This shift was also detected for vaccinees and could be explained by the amount of lower-quality studies that included disease risk attributes (Figure 1). No changes occurred among the domains that were least reported statistically significant.

**Table 2 Overview of all studies reporting relative statistical significance of domains (representatives)**

| Category & domain (n=)* | Statistical significance** | | | | | | |
| --- | --- | --- | --- | --- | --- | --- | --- |
|  | **P<0.10** | **P<0.05** | **P<0.01** | **P<0.001** | **P<0.0001** | **Tot.** | **Not sign.** |
| **Outcome** |  |  |  |  |  |  |  |
| Protection duration (n=7) | 0 | 2 | 3 | 2 | 0 | 7 | 1 |
| Vaccine effect. (n=14) | 0 | 8 | 5 | 2 | 1 | 16 | 0 |
| Vaccine risk (n=17) | 1 | 10 | 3 | 4 | 1 | 19 | 1 |
| **Process** |  |  |  |  |  |  |  |
| Dosing & visits (n=6) | 0 | 3 | 1 | 1 | 0 | 5 | 2 |
| Service delivery (n=3) | 0 | 4 | 0 | 0 | 0 | 4 | 2 |
| Target group (n=2) | 0 | 1 | 1 | 0 | 0 | 2 | 0 |
| Time (n=3) | 0 | 0 | 3 | 1 | 0 | 4 | 0 |
| Vaccination age (n=1) | 0 | 1 | 0 | 0 | 0 | 1 | 0 |
| Vaccine accessibility (n=2) | 0 | 2 | 0 | 0 | 0 | 2 | 0 |
| Vaccine adm. (n=2) | 0 | 0 | 2 | 0 | 0 | 2 | 1 |
| Vaccine content (n=3) | 0 | 1 | 1 | 1 | 0 | 3 | 0 |
| **Cost** |  |  |  |  |  |  |  |
| Cost (n=15) | 0 | 5 | 7 | 3 | 1 | 16 | 1 |
| **Other** |  |  |  |  |  |  |  |
| Context (n=5) | 0 | 5 | 1 | 1 | 0 | 7 | 0 |
| Disease risk (n=6) | 0 | 7 | 1 | 0 | 0 | 8 | 1 |
| Information (n=2) | 0 | 0 | 3 | 0 | 0 | 3 | 1 |
| Other (n=5) | 0 | 3 | 2 | 1 | 0 | 6 | 0 |

*n=number of studies reporting a particular domain. 21/27 studies reported statistical significance and/or attribute p-values; ** *Information is based on main models and pooled data when available (if not, data of separate models/classes was used). Some studies included more than one attribute related to a particular domain. Totals could hence exceed the total number of studies incorporated.*

*Overall preferences and comparison of vaccinees and representatives*

High- and lower-quality studies used 243 attributes in total, of which 39.1% were classified as outcome, 24.3% as process, 12.8% as cost and 23.9% as other. Compared to high-quality studies, two domains were introduced by lower-quality studies (vaccine content and other). Four of the five most popular attributes among high-quality studies were also most frequently reported among all studies (Figure 1). As shown in Additional file 6, minor sequence changes took place by including lower-quality studies. Outcome measures vaccine effectiveness and vaccine risk changed places (rank 1 and 2) as well as protection duration and disease risk (rank 4 and 6). In line with the high-quality studies, process-related domains were least reported (esp. target group).

Statistical significance was reported (or could be determined) in 38 studies and was defined by five different alphas ranging from p<0.10 to p<0.0001 (Additional file 7). All domains that were most and least commonly statistically significant were in correspondence with high-quality studies, except for disease risk (other) and service delivery (process).

**Figure 1 Frequency of domains among all studies**

**References**

1.Verelst F, Kessels R, Delva W, Beutels P, Willem L. Drivers of vaccine decision-making in South Africa: A discrete choice experiment. Vaccine. 2019;37(15):2079-89.

2. Marshall HS, Chen G, Clark M, Ratcliffe J. Adolescent, parent and societal preferences and willingness to pay for meningococcal B vaccine: A discrete choice experiment. Vaccine. 2016;34(5):671-7.
